# HIV-1 requires capsid remodelling at the nuclear pore for nuclear entry and integration

**DOI:** 10.1371/journal.ppat.1009484

**Published:** 2021-09-20

**Authors:** Anabel Guedán, Callum D. Donaldson, Eve R. Caroe, Ophélie Cosnefroy, Ian A. Taylor, Kate N. Bishop

**Affiliations:** 1 Retroviral Replication Laboratory, The Francis Crick Institute, London, United Kingdom; 2 Macromolecular Structure Laboratory, The Francis Crick Institute, London, United Kingdom; University of North Carolina at Chapel Hill, UNITED STATES

## Abstract

The capsid (CA) lattice of the HIV-1 core plays a key role during infection. From the moment the core is released into the cytoplasm, it interacts with a range of cellular factors that, ultimately, direct the pre-integration complex to the integration site. For integration to occur, the CA lattice must disassemble. Early uncoating or a failure to do so has detrimental effects on virus infectivity, indicating that an optimal stability of the viral core is crucial for infection. Here, we introduced cysteine residues into HIV-1 CA in order to induce disulphide bond formation and engineer hyper-stable mutants that are slower or unable to uncoat, and then followed their replication. From a panel of mutants, we identified three with increased capsid stability in cells and found that, whilst the M68C/E212C mutant had a 5-fold reduction in reverse transcription, two mutants, A14C/E45C and E180C, were able to reverse transcribe to approximately WT levels in cycling cells. Moreover, these mutants only had a 5-fold reduction in 2-LTR circle production, suggesting that not only could reverse transcription complete in hyper-stable cores, but that the nascent viral cDNA could enter the nuclear compartment. Furthermore, we observed A14C/E45C mutant capsid in nuclear and chromatin-associated fractions implying that the hyper-stable cores themselves entered the nucleus. Immunofluorescence studies revealed that although the A14C/E45C mutant capsid reached the nuclear pore with the same kinetics as wild type capsid, it was then retained at the pore in association with Nup153. Crucially, infection with the hyper-stable mutants did not promote CPSF6 re-localisation to nuclear speckles, despite the mutant capsids being competent for CPSF6 binding. These observations suggest that hyper-stable cores are not able to uncoat, or remodel, enough to pass through or dissociate from the nuclear pore and integrate successfully. This, is turn, highlights the importance of capsid lattice flexibility for nuclear entry. In conclusion, we hypothesise that during a productive infection, a capsid remodelling step takes place at the nuclear pore that releases the core complex from Nup153, and relays it to CPSF6, which then localises it to chromatin ready for integration.

## Author summary

The mature viral core of human immunodeficiency virus (HIV) consists of a highly organised lattice formed by capsid molecules that encloses the viral RNA and viral enzymes. This lattice is crucial during the early stages of viral replication, as it has to break down–uncoat–at the right time and place in order for the viral DNA to integrate successfully. Lentiviruses, like HIV, can infect non-dividing cells and are able to access the host cell DNA by entering the nucleus through nuclear pores. Until recently, uncoating was thought to occur in the cytoplasm as the whole core was thought too large to pass through the nuclear pore. However, lately it has been suggested that uncoating might occur at the nuclear pore or even inside the nucleus and the site of uncoating is currently hotly debated. By investigating HIV mutants with an increased lattice stability, we have shown that lattice flexibility is crucial for nuclear entry. Provocatively, we observed hyper-stable mutant capsid in nuclear and chromatin-associated fractions suggesting that uncoating is not required for nuclear entry. Nonetheless, microscopy experiments suggested that these hyper-stable mutants were retained on the inner side of the nuclear pore, and were impaired for downstream events in the nucleus, including CPSF6 accumulation in nuclear speckles, leading to a severe infectivity defect. Therefore, we believe that an essential uncoating, or capsid lattice remodelling event normally takes place at the nuclear pore.

## Introduction

Upon infection of the target cell, the human immunodeficiency virus 1 (HIV-1) core is released into the cytoplasm before trafficking to the nucleus. During this process, the viral RNA genome is reverse transcribed by the viral reverse transcriptase (RT) into double-stranded DNA forming what is known as the reverse transcription complex (RTC). Inside the nucleus, the viral DNA is the main component of the pre-integration complex (PIC) and is integrated by the viral integrase (IN) into the host cell genome to form a provirus [1].

The mature core of HIV-1 consists of a highly organised lattice of capsid (CA) molecules, encasing the viral RNA genome and associated viral proteins. The lattice is composed of approximately 1500 CA monomers assembled into about 250 hexamers and exactly 12 pentamers, forming a fullerene cone shape [2–5]. The CA monomer is a largely helical protein with two independent folded domains separated by a flexible linker: the N-terminal domain (NTD) and the C-terminal domain (CTD) [2,6–8]. Structural studies have provided key information on how the CA lattice is organised [8–14]. Intra-hexamer and inter-hexamer interactions at different lattice interfaces contribute to an optimal CA lattice stability. The intra-hexamer interactions include NTD-NTD and NTD-CTD interactions between adjacent CA monomers within a hexamer, whereas the inter-hexamer interactions include dimeric and trimeric CTD-CTD interactions between neighbouring hexamers [11,14].

As the outer surface of the viral core, the CA lattice protects the viral DNA from cytosolic sensors [15,16] but it can be recognised by cellular restriction factors that inhibit viral replication [17,18]. Moreover, from cell entry to integration, the CA lattice interacts with many cellular factors. Some of these are cytoplasmic, including cyclophilin A (CYPA) [19–21], the cellular motors dynein and kinesin-1 [1,22,23] and FEZ1 [24]. Others shuttle between the cytoplasm and the nucleus like transportin-3 (TNPO3) [25–27] and transportin-1 [28]. There are further interactions at the nuclear envelope with nuclear pore complex (NPC) proteins, particularly nucleoporins 358 (NUP358) and 153 (NUP153) [25,26,29–31] and with nuclear proteins such as cleavage and polyadenylation factor 6 (CPSF6); [31–35]. The roles of most of these cellular factors in viral replication are not yet fully understood.

Prior to integration, the CA lattice is assumed to disassemble in a process termed uncoating; however, the timing, location and even the exact definition of this process remains unclear. For many years, uncoating was believed to occur immediately after viral entry [36,37] but, more recently, data has suggested that it might occur after a short time in the cytoplasm, at the nuclear pore or even, most recently, inside the nucleus [38–44]. Indeed, there is growing evidence to suggest that at least some CA is present in the nucleus, although its oligomeric state and function have yet to be fully characterised [34,40–48]. For this reason, using the term “remodelling” may be more appropriate than uncoating to suggest changes in the CA lattice. We, and others, have also reported a link between CA loss and reverse transcription [38,49–51]. Uncoating is inhibited if reverse transcription is stalled. Initially, this was taken as evidence for cytoplasmic uncoating [49]. However, when combined with emerging data suggesting CA can reach the nucleus more rapidly than previously thought [43,44,48], it has reopened the debate about where reverse transcription is actually completed during infection, and so does not currently preclude any specific uncoating model.

Importantly, the location and timing of uncoating together with optimal stability of the CA lattice seem to be key to successful infection. Early uncoating, or a failure to uncoat, both have detrimental effects on viral infectivity [15,52,53], making the CA lattice an attractive target for drug development [54,55]. Here we set out to investigate the effects of increased core stability on the early stages of HIV-1 infection. To this end, we introduced mutations into CA predicted to stabilise the mature CA lattice at different interfaces. We show that mutations that create a hyper-stable lattice reduce virus infectivity by inhibiting integration, but only slightly impede reverse transcription. Analysis of CA protein levels within different subcellular fractions during infection revealed higher levels of hyper-stable mutant CA in all fractions over time, including in the nucleus. Finally, immunofluorescence data suggest that the hyper-stable mutant CA lattice is retained around the nuclear pore and that it is unable to promote CPSF6 re-localisation to nuclear speckles. Based upon these observations, we propose a model where hyper-stable mutants are unable to uncoat or “remodel” their capsid lattice to the required extent to successfully deliver the viral DNA for integration into the host cell genome.

## Results

### Cysteine mutations at different CA lattice interfaces are able to stabilise the viral core in cells

**Fig 1:**
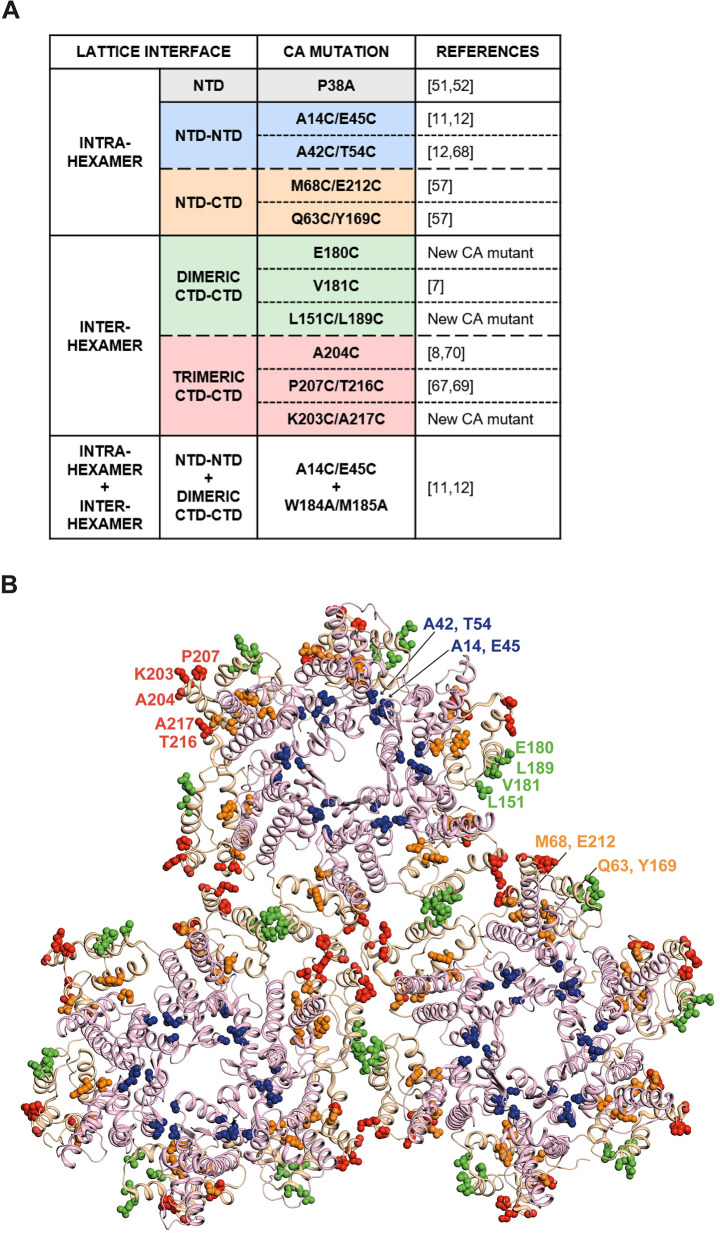
Panel of CA mutants. (A) The panel of CA mutants used in this study and the lattice interface at which the mutations reside. New CA mutants with cysteine mutations (E180C, L151C/L189C and K203C/A217C) were designed based on a previously published cryo-EM MDFF atomic model (PDB ID: 3J34; [8]) and crystal structures (PDB ID: 3H4E and 3H47; [11]). The remaining CA mutants were selected from previous publications as indicated in the references column. (B) Structure of the CA lattice from PDB ID: 3J34, showing 18 CA monomers arranged into three hexameric rings. The protein backbone is shown in cartoon representation with CA-NTDs coloured pink and CA-CTDs coloured wheat. Residues at intra- and inter-hexamer interfaces where mutations were made are highlighted in space-fill representation and colour-coded according to the interaction type displayed in (A).

Previous *in vitro* studies introduced cysteine mutations at the CA NTD-NTD interface to create disulphide bridges in order to stabilise CA hexamers for crystallisation [11,12]. We decided to use the same approach to examine if similar mutations would increase the stability of the CA lattice during infection of cells, to investigate the effect of core stability on the early stages of HIV-1 infection. We selected CA mutants from the literature and identified additional CA residues to substitute with cysteine residues in order to stabilise all of the different inter-hexamer interfaces with disulphide bridges (listed in Fig 1A and residues highlighted in Fig 1B, colour coded for lattice interface). The new mutants E180C, L151C/L189C and K203C/A217C were designed based on a previous report demonstrating that disulphide bonds can have a variable Cβ-Cβ inter-residue spacing of between 3.5–4.5Å [56]. Thus, employing a cryo-EM MDFF atomic model of an *in vitro* CA tubular assembly [PDB ID: 3J34; [8]] and crystal structures [PDB ID: 3H4E and 3H47; [11]], we selected residue pairs with a Cβ-Cβ distance within this range at the various interfaces for site-directed mutagenesis. Consequently, we created a panel with at least two mutants at each CA lattice interface. In addition, the P38A mutant was included in the panel as a negative control, as it has shown to be less stable than WT [51,52]. The original mutant used to determine the crystal structure of a hexameric CA assembly, A14C/E45C/W184A/M185A, [11,12] was also included as a negative control.

Firstly, we synthesised viral-like particles (VLP) containing the different CA mutants and a GFP reporter gene. We assessed VLP production by measuring the RT activity in the supernatants of producer 293T cells using a modified RT ELISA. As expected, the A14C/E45C/W184A/M185A and W184A/M185A mutants that weaken CTD-CTD interactions were severely impaired for virus production (S1A Fig). We confirmed that this was not due to a lack of Gag expression of these mutants, by immunoblotting 293T producer cell lysates with an anti-HIV-1 CA antibody and showing that the level of Gag expression in the mutants was similar to WT (S1B Fig). Most of the mutants, however, showed similar titres to WT VLP, implying that these mutations did not affect Gag assembly. Of note, the inter-hexamer mutants V181C, L151C/L189C and K203C/A217C were partially impaired for VLP production (S1A Fig), likely due to the importance of this interface on virus assembly [3,53]. Whilst we confirmed that expression of these mutant capsid proteins in 293T producer cell lysates was similar to WT VLP (S1C Fig), we detected an additional, smaller, CA band for both L151C/L169C and K203/A217C. This has been reported previously [12,57] and probably represents a processing defect, such as inappropriate cleavage of CA by the viral protease due to the structural changes [57]. This may be the cause of the reduced viral titres. Thus, based on these results, the L151C/L189C and the K203C/A217C mutants as well as the A14C/E45C/W184A/M185A and W184A/M185A mutants were not included in further experiments.

**Fig 2:**
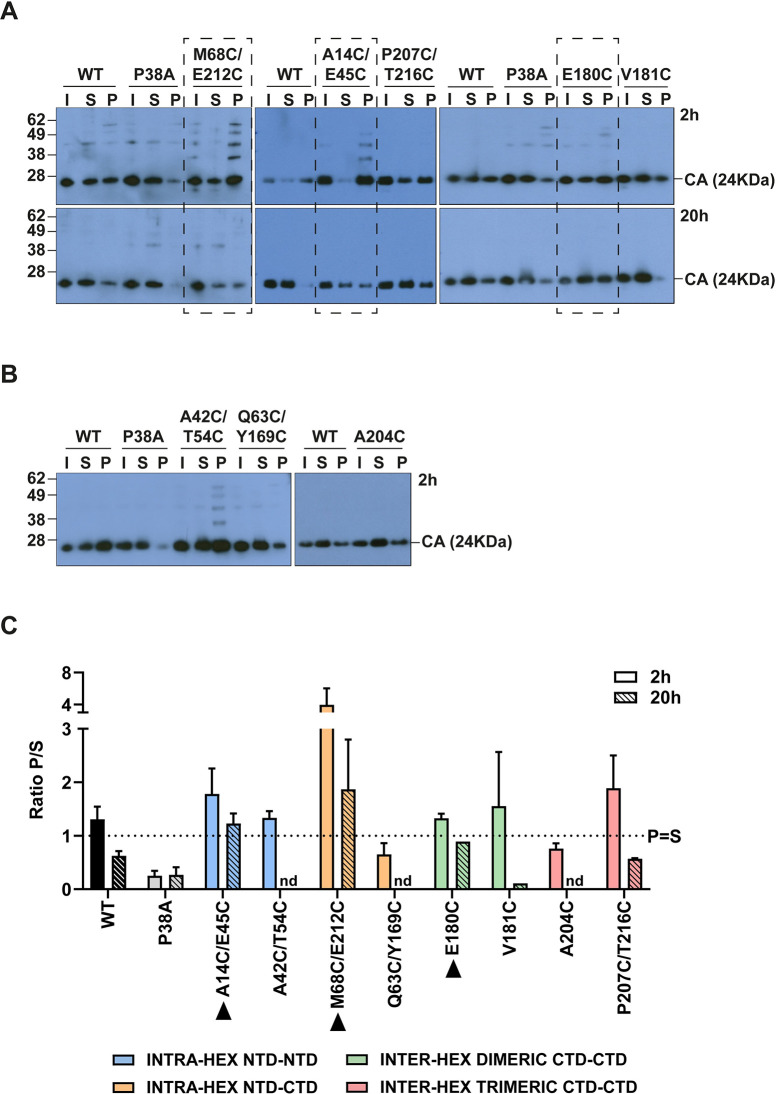
Effect of the CA mutations on viral core stability in cells. (A, B) Representative immunoblots of Fate-of-capsid assays comparing WT VLP to mutant VLP. HeLa cells were infected with equal RT units of WT or mutant VLP and cell lysates were harvested at 2 hpi (A, B) and 20 hpi (A). Cell lysates were centrifuged through a sucrose cushion to separate viral CA into free (soluble, S) and assembled (pellet, P) fractions. An input (I) sample was also harvested before the centrifugation through the sucrose cushion. CA was detected by immunoblotting using an anti-HIV-1 CA antibody. Each assay was performed at least three times independently. (C) Bar chart summarising the densitometry analysis of independent immunoblots of Fate-of-capsid assays. Data is plotted as a mean of the ratio P/S ± SEM. The dotted line indicates a ratio of 1, when P = S. nd = not determined. Bars are colour coded according to the lattice interface at which the cysteines have been introduced, as in Fig 1. Mutant VLP with a CA ratio of P>S at 2h and P = S at 20h were considered “hyper-stable” (surrounded with a dashed-line box in (A) and indicated with black arrow heads in (C)).

Next, we performed fate-of-capsid assays, to study the effect of the cysteine mutations on viral core stability in cells (Fig 2). HeLa cells were infected with equal titres of WT and mutant VLP, and cell lysates were harvested at 2 and 20 hours post-infection (hpi). These time points were selected as they have previously been used to monitor the stability of the viral core at the early and late stages of the virus replication cycle [17]. The cell lysates were centrifuged through a sucrose cushion to be separated into two fractions: free or soluble CA (S) and pelleted CA (P), which contains assembled cores [58]. An input (I) sample was also harvested before centrifugation through the sucrose cushion. The fractions were analysed by immunoblotting with an anti-HIV-1 CA antibody and the ratio of pelleted to soluble CA was determined. Fig 2A and 2B show representative blots of the fate-of-capsid assay for each mutant. A WT sample was included in every assay for comparison. Although there was some variation between assays, the WT samples showed similar amounts of CA in the pellet and soluble fractions (approximately P = S) at 2 hpi and less CA in the pellet (P<S) at 20hpi (Fig 2C), presumably reflecting a reduction in the amount of assembled CA with time due to uncoating. In agreement with previous reports, we recovered much less CA in the pellet fraction than the supernatant fraction from cells infected with the negative hypo-stable control, P38A [51,52]. For this mutant, P<S at both 2 and 20 hpi (Fig 2C), indicating the small amount of assembled CA at both time points. Therefore, to determine which mutants had more stable CA lattices than WT VLP, we set a CA ratiometric profile criteria for mutants to be considered hyper-stable as the following: Mutants should consistently show greater amounts of CA in the pellet than in the supernatant (P>S) at 2 hpi and approximately equal amounts in both fractions (P = S) at 20 hpi. Three mutants showed this phenotype: M68C/E212C, A14C/E45C and E180C (Fig 2C, black arrows and highlighted with dashed boxes in Fig 2A). Although P207C/T216C and the V181C mutants appeared more stable than WT at 2 hpi, they were not more stable at 20 hpi (Fig 2C), suggesting that neither of these mutants had increased viral core stability compared to WT. Thus, taken together despite the cytoplasm being a reducing environment [59,60], these data showed that three of the panel of cysteine mutants had increased core stability in cells and could be used to study the effects of stabilizing the viral core on replication.

### Most CA mutants are less infectious, regardless of CA lattice stability

**Fig 3:**
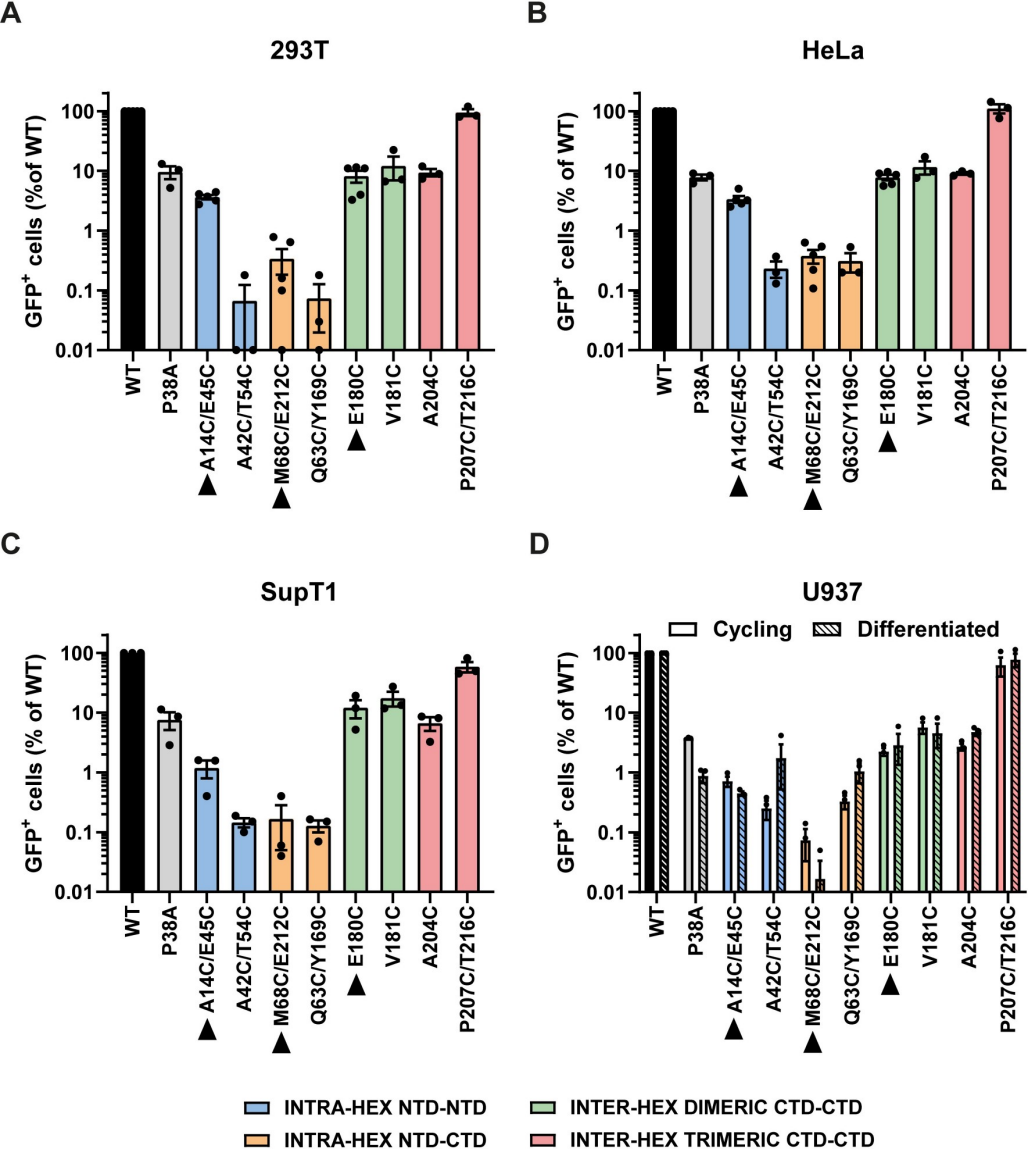
Effect of CA mutations on VLP infectivity. 293T (A), HeLa (B), SupT1 (C) and both cycling and differentiated U937 (D) cells were infected with equal RT units of GFP-reporter WT or mutant VLP. The percentage of GFP+ cells was measured by flow cytometry at 72hpi and plotted relative to WT VLP. Points indicate individual biological repeats and lines show the mean ± SEM. Bars are colour coded according to the lattice interface at which the cysteines have been introduced, as in Fig 1. Hyper-stable mutants based on the fate of capsid assay in Fig 2 are indicated with black arrow heads.

To investigate the effect of the CA mutations on virus infectivity, we infected four cell lines (293T, HeLa, SupT1 and U937 cells) with equal RT units of VSVg-pseudo-typed GFP-reporter WT or mutant VLP and analysed the percentage of GFP+ cells at 72 hpi by flow cytometry (Fig 3). As observed previously, the P38A mutant that had reduced CA lattice stability had a marked reduction in GFP expression in all the cell lines (Fig 3, grey bars). Apart from the inter-hexamer P207C/T216C mutant that showed similar infectivity to WT, the remainder of the mutants also showed decreased infectivity, ranging from ~0.05–10% of WT infectivity. A similar pattern of infectivity was seen across all the cells lines indicating that there were no cell-type specific effects, with generally lower overall infectivity in U937 cells. Differentiating the U937 cells did not alter the levels of viral infectivity (Fig 3D), implying that these effects were not linked to the cell cycle and virions were not impeded further in non-cycling cells. Interestingly, the level of infectivity did not correlate with the CA lattice stability determined by the fate-of-CA assay (Fig 2) but there was some correlation with the CA interface modified (see colour coding Figs 1A and 3), with the intra-hexamer mutants (blue and orange bars) being more defective than the inter-hexamer mutants (green and red bars). Of the three hyper-stable mutants, A14C/E45C, M68C/E212C and E180C (Fig 3, black arrowheads), the infectivity ranged from 0.7–4%, 0.07–0.4% and 2–10% respectively between the different cell lines.

### Reverse transcription can complete in hyper-stable cores but infectivity is severely inhibited

**Fig 4:**
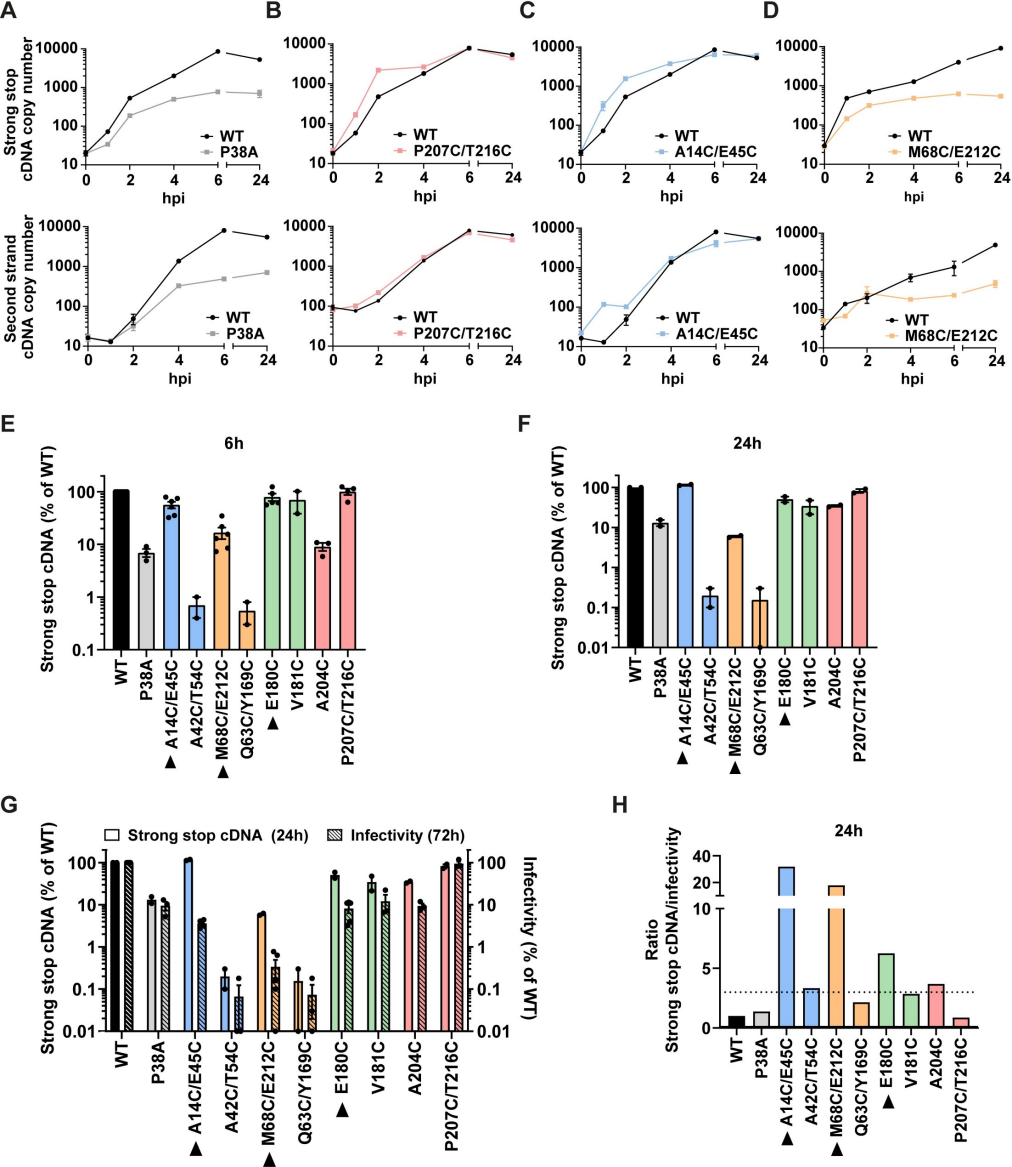
Effect of CA mutations on reverse transcription. 293T cells were synchronously infected with equivalent RT units of WT or mutant VLP. At the indicated times post-infection, cells were harvested for DNA extraction, and viral cDNA products were measured by qPCR. (A-D) Graphs show the levels of early (strong stop) cDNA (upper panels) and late (second strand) cDNA (lower panels) in 293T cells following infection with WT VLP (black line) and mutants P38A (A), P207C/T216C (B), A14C/E45C (C) or M68C/E212C (D). The data is shown as mean ± SEM from at least two independent experiments. (E, F) Bar charts show the levels of strong stop cDNA at 6h (E) and 24h (F) post infection for the panel of mutants relative to WT infections. (G) Bar chart showing the levels of strong stop cDNA at 24 h (left y-axis) and infectivity (from Fig 3) at 72 h (right y-axis) compared to WT VLP for each mutant. Individual points represent biological repeats and lines indicate the mean ± SEM. (H) Bar chart showing the ratio of relative levels of strong stop cDNA to infectivity, from (G). Dashed line indicates a ratio of 3. Bars are colour coded according to the lattice interface at which the cysteines have been introduced, see Fig 1. Hyper-stable mutants are indicated with a black arrow heads.

Next, to determine which step in replication was affected by the CA mutations, we examined the ability of each mutant to reverse transcribe (Fig 4). 293T cells were synchronously infected with equal RT units of WT or mutant VLP, and at 0, 1, 2, 4, 6 and 24 hpi, cells were harvested and the DNA extracted and analysed for early (strong stop) and late (second strand) viral cDNA products by qPCR (Fig 4). Following infection with WT VLP, the amount of viral cDNA products increased with time, peaking at 6 hpi (Fig 4A–4D, black line). As seen before, infection with mutant P38A resulted in less accumulation of viral cDNA (Fig 4A, grey lines). This ~90% decrease in cDNA accumulation mirrored the 90% decrease in infectivity (Fig 3). Likewise, the P207C/T216C mutant had similar infectivity to WT VLP and had similar levels of reverse transcription (Fig 4B, red lines). However, the hyper-stable mutants A14C/E45C and M68C/E212C had a different phenotype. Surprisingly, despite a ~95% decrease in infectivity, A14C/E45C reverse transcribed to WT levels (Fig 4C, blue lines) and mutant M68C/E212C still produced about 10% the cDNA of WT (Fig 4D, orange lines), even though its infectivity was reduced to less than 1% (Fig 3). Fig 4E and 4F show a summary of the amount of strong stop cDNA accumulated at 6 and 24 hpi, respectively, for all the mutants. The E180C and V181C mutants showed WT levels of strong stop cDNA, A204C accumulated about 10% the cDNA of WT and A42C/T54C and Q63C/Y169C only accumulated ~1% the cDNA of WT. Similar results were obtained when looking at the levels of second strand cDNA (S2A Fig). In general, the level of cDNA accumulation at 24 hours compared to WT correlated with relative level of infectivity compared to WT (Figs 4G and S2B). To visualise this correlation, we plotted these data as a ratio of relative cDNA levels to relative infectivity. Most mutants had a ratio of less than 3 (Figs 4H and S2C). Therefore, it can be assumed that, in general, the reduction in infectivity results from the effect on reverse transcription. The only mutants where the level of reverse transcription products did not correlate with the level of infectivity was for the three hyper-stable mutants (Figs 4H and S2C). These data suggest that even though increasing core stability has detrimental effects on infectivity, the block is after reverse transcription. Therefore, although reverse transcription promotes uncoating of WT virus [38,49–51,61], reverse transcription is not dependant on uncoating.

**Fig 5:**
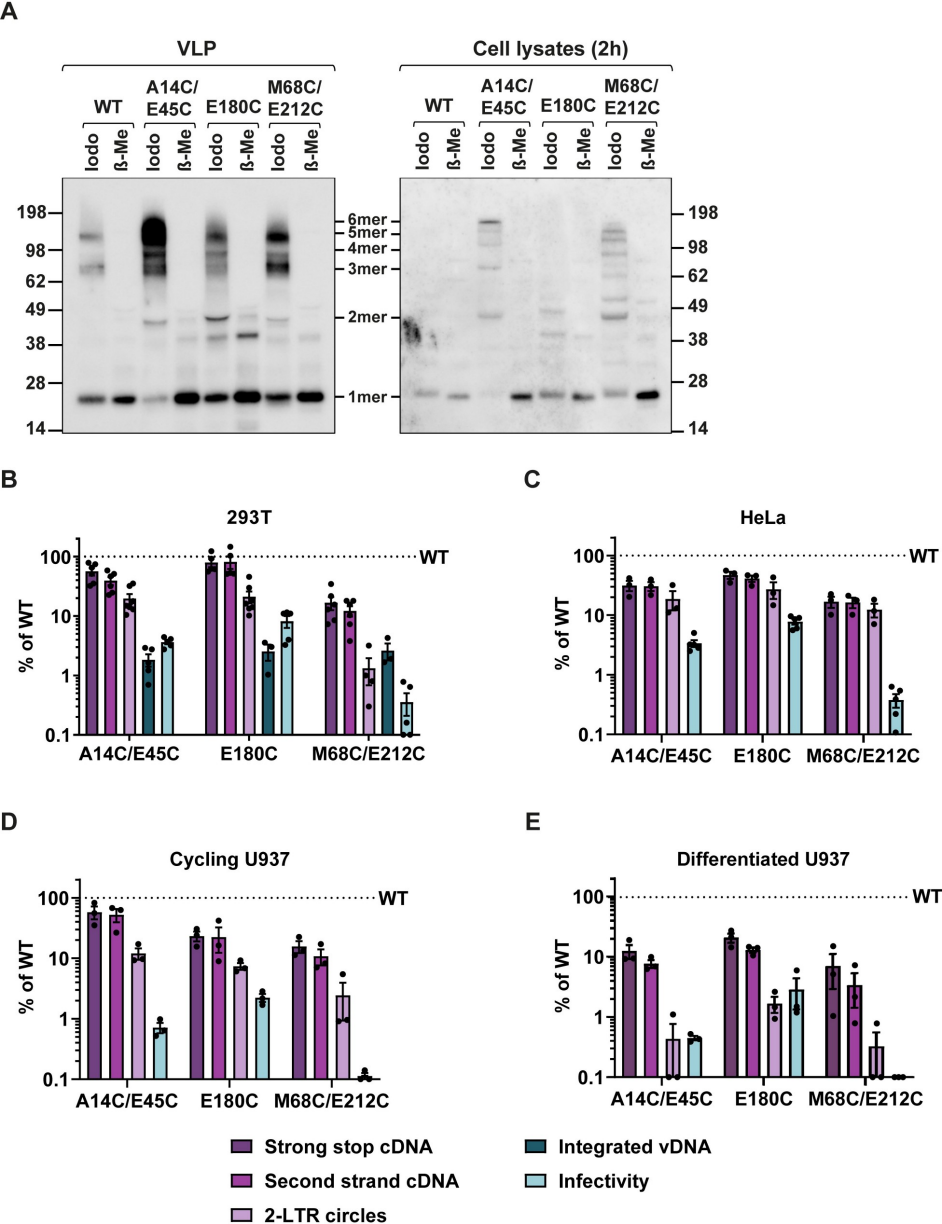
Effect of hyper-stable CA mutations on the early stages of infection. (A) Disulphide cross-linking of CA monomers in VLP and cells. Equal RT units of WT VLP or hyper-stable mutants A14C/E45C, E180C or M68C/E212C VLP were pelleted by centrifugation and resuspended in Laemmli buffer. VLP were either analysed directly or used to infect HeLa cells. 2 hpi cells were lysed and both VLP and cell lysates were analysed by non-reducing SDS-PAGE and immunoblotting with an HIV-1 CA antibody. Samples were either treated with 50mM iodoacetamide (Iodo), to prevent further disulphide bond formation, or with 10% β-Mercaptoethanol (β-Me), to reduce existing disulphide bonds, prior to SDS-PAGE. The expected band positions of monomeric (1mer) and different oligomeric CA forms (2mer, 3mer etc) are indicated. (B) 293T, (C) HeLa, (D) cycling U937 and (E) differentiated U937 cells were synchronously infected with equivalent RT units of WT or mutant VLP and harvested for DNA extraction at the following times: 293T, HeLa and cycling U937 cells were harvested at 6hpi to measure early (strong stop) and late (second strand) cDNA and at 24hpi to measure 2-LTR circles. 293T cells were harvested at 2 weeks post infection to measure integrated proviral DNA by qPCR. Differentiated U937 cells were harvested at 24h to measure early and late cDNA and at 72hpi to measure 2-LTR circles by qPCR. Data were plotted relative to WT infections (shown as a dashed line at 100%). Points indicate individual biological repeats and the mean ± SEM are shown. Viral infectivity from parallel infections (see Fig 3) was also plotted for comparison.

We therefore decided to focus on the A14C/E45C, E180C and M68C/E212C mutants to investigate the effect of core stability on the other early replication steps. First, we examined whether the observed increase in core stability of these mutants was indeed due to disulphide bridge formation as designed, and not due to other stabilising effects of the individual mutations. HeLa cells were infected with equal RT units of WT and mutant VLP and cell lysates were harvested at 2 hpi. Both the input VLP lysates and infected HeLa cell lysates were then analysed by SDS-PAGE in reducing or non-reducing conditions followed by immunoblotting with an anti-HIV-1 CA antibody (Fig 5A). Samples were either treated with iodoacetamide (Iodo) to prevent artefactual cysteine formation (non-reducing conditions), or β-mercaptoethanol (β-ME) to reduce disulphide bonds (reducing conditions) prior to SDS-PAGE. Disulphide cross-linking of CA monomers was detected in the non-reducing conditions for all three hyper-stable mutants in both VLPs and infected cells. The proportion of oligomeric CA to monomeric CA was similar in virions and cells. Although some higher order oligomers were detected in WT virions in non-reducing conditions, only monomeric CA of 24KDa was detected in cells infected with WT VLP in non-reducing conditions. Upon addition of β-ME, most of the higher molecular weight bands disappeared in both virions and cells, suggesting that intra- or inter-hexamer disulphide bonds form in virions during maturation and are maintained when these particles infect cells. Therefore, it seems likely that they contribute to the increased core stability of these mutants. A minor band was detected in all samples (but strongest in the E180C virions under reducing conditions) at approximately 43KDa. As this band was present in both reducing and non-reducing conditions it is presumably not formed by disulphide linkages and may instead be a processing intermediate (possibly the MA-p2-CA fragment).

To further investigate the infectivity block to the hyper-stable mutants, we measured the ability of the mutants to complete different steps of replication between reverse transcription and integration in a variety of different cell lines. Following synchronous infection with equal RT units of WT or mutant VLP, samples were taken to measure levels of early and late cDNA, 2-LTR circles and integrated viral DNA by qPCR. It is widely accepted that 2-LTR circles are an indicator of completion of reverse transcription and nuclear entry. Fig 5B shows the relative amounts of the different viral DNA products compared to WT VLP in 293T cells. Interestingly, as well as being only slightly defective for reverse transcription, both A14C/E45C and E180C mutant VLP were able to produce ~20% the amount of 2-LTR circles produced by WT VLP. This suggests that at least some of these mutant particles can enter the nucleus. Importantly, these mutants integrated <2% of the amount of cDNA integrated in WT infections, which correlated well with the decrease in infectivity measured. Therefore, there appears to be a minor block to nuclear entry for these two mutants, but, surprisingly, the major defect in these cells is after nuclear entry and before integration. In contrast, mutant M68C/E212C VLP only produced ~10% of the cDNA of WT, and there was a further reduction in the amount of 2-LTR circles, to <1% of that produced by WT VLP. There was no further decrease in integration compared to the levels of 2-LTR circles, suggesting that the M68C/E212C mutant is more severely impaired earlier in replication than the other two hyper-stable mutants and is blocked for nuclear entry. Interestingly, the M68C/E212C appeared to be the most stable mutant in our fate of CA assays (Fig 2). Fig 5C, 5D and 5E show the relative amounts of the different viral DNA products compared to WT VLP in HeLa cells, cycling monocytic U937 cells, and differentiated U937 cells respectively. Like in 293T cells, all three mutants were able to reverse transcribe to at least ~20% of WT levels in HeLa and cycling U937 cells (Fig 5C and 5D), although the levels dropped to ~10% in differentiated U937 cells for all mutants (Fig 5E). There was a further decrease in the amount of 2-LTR circles compared to WT in cycling U937 cells (Fig 5D), although this was less pronounced in HeLa cells (Fig 5C). However, there was a greater reduction in infectivity (Fig 5C and 5D). This confirms that the hyper-stable mutants have a major block to replication after 2-LTR synthesis in cycling cells. In differentiated U937 cells, the levels of 2-LTR circles dropped to below 1% that of WT virions which correlated with the reduction in infectivity (Fig 5E). This suggests that the mutants are inhibited slightly earlier in infection in these cells and that nuclear entry is blocked. Recent studies [44] suggest that reverse transcription likely finishes in the nucleus in macrophages which may explain the reduction in reverse transcription observed in differentiated U937 cells. In every cell line, the M68C/E212C mutant was the most severely impaired at each replication stage. Altogether, these data suggest that it is possible for reverse transcription to finish in a hyper-stable core (because 2-LTR circles can only be synthesised from full length cDNA) and that at least some of this cDNA is then able to enter the nuclear compartment (where the ligases needed for 2-LTR synthesis are presumably located) in cycling cells, but that even when reverse transcription does occur, there is a block to integration that prevents these hyper-stable mutants successfully forming proviruses.

### CA protein from the A14C/E45C mutant is detected in nuclear and chromatin fractions

**Fig 6:**
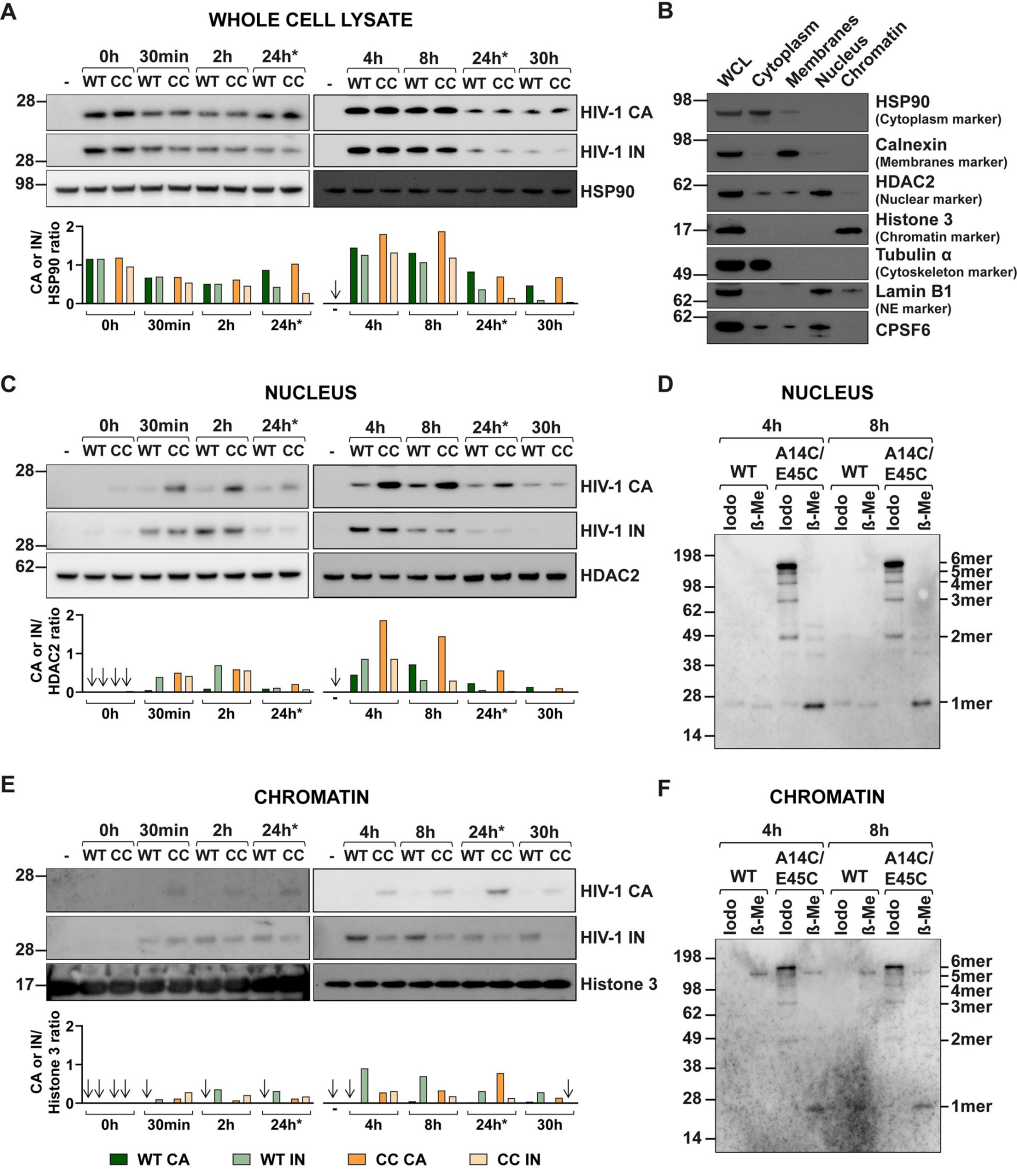
Cellular localisation of CA and IN proteins during WT and A14C/E45C infections. HeLa cells were synchronously infected with equal RT units of WT or A14C/E45C mutant (CC in the figure) VLP. Cells were harvested at either 0, 0.5 and 2 hpi, or 4, 8, 24 and 30 hpi to be processed in parallel as a whole cell lysate (WCL) or to undergo subcellular fractionation. Protein levels were quantified by BCA assay, proportional amounts of the fractions related to the WCL were loaded on SDS-PAGE gels and analysed by immunoblotting using the following antibodies: Anti-CA and anti-IN for HIV-1 proteins, anti-HSP90 for cytoplasm, anti-calnexin for membranes, anti-HDAC2 for nucleus, anti-histone 3 for chromatin and anti-tubulin α for cytoskeleton. Anti-lamin B1 was used as a nuclear envelope marker and the distribution of CPSF6 was also analysed. (A, C, E) Panels show representative immunoblots probed for HIV-1 CA and IN and the appropriate fractionation marker (A) WCL with HSP90 as a loading control, (C) Nuclear fraction with HDAC2 as a loading control. (E) Chromatin fraction with histone 3 as a loading control. “-”indicates uninfected cells. Bar charts show the densitometry analysis of the immunoblots plotted as the ratio of CA or IN proteins to the loading control. The key for bar chart colour coding is shown at the bottom of the figure. Arrows indicate undetectable protein. The 24 hpi sample was loaded on each gel for the sake of comparison (24h*). (B) Subcellular fractions from uninfected cells, probed for control proteins to confirm successful sample fractionation. (D, F) Disulphide cross-linking of CA monomers in different cellular fractions. The 4 and 8hpi samples from nucleus (D) and chromatin (F) fractions were either treated with 50mM iodoacetamide (Iodo), to prevent further disulphide bond formation, or with 10% β-Mercaptoethanol (β-Me), to reduce existing disulphide bonds, prior to SDS-PAGE and immunoblotting with an anti-HIV-1 CA antibody. The expected band positions of monomeric (1mer) and different oligomeric CA forms (2mer, 3mer etc) are indicated.

Historically, the nuclear pore has been considered too small to allow passage of a complete HIV core. However, very recent work has revealed the pore to be larger than previously measured and actually wider than the core, which may change our ideas of the role of CA in nuclear entry [62,63]. We have shown here that hyper-stable CA mutants can synthesise cDNA and that at least some of this cDNA can reach the nucleus to form 2-LTR circles. This suggests that either the cDNA can escape the core in order to enter the nucleus, despite the lattice being more resistant to disassembly, or that the DNA-containing core can enter the NPC. Therefore, in order to determine the cellular localisation of components of a hyper-stable core, we measured CA and IN protein levels in different cellular compartments during infection (Fig 6). HeLa cells were synchronously infected with equal RT units of WT or A14C/E45C VLP and cells were harvested at either 0, 0.5 and 2 hpi or at 4, 8, 24 and 30 hpi in different experiments. The 24 hpi samples were run on every blot to check exposure levels. Viral proteins detected at 0 hpi represent VLP attached to cells during spinoculation (Fig 6A). Although there was some variation in protein levels between different time points, importantly, the total levels of both CA and IN in whole cell lysates were similar between WT and A14C/E45C (CC) infection at all time points (Figs 6A and S3). The infected cell lysates were further processed into the following subcellular fractions: cytoplasm, membranes, nucleus and chromatin-bound. The separation resolution of the subcellular fractionation was determined by looking for distinct markers in the different fractions: HSP90 for the cytoplasm, calnexin for the membranes, HDAC2 for the nucleus and histone 3 for chromatin-bound. The representative immunoblot in Fig 6B shows that the fractionation procedure was highly effective, and the markers were predominantly found in the expected fractions. There was a little of the nuclear markers HDAC2 and CPSF6 detected in the cytoplasmic and membrane fractions but this was minor contamination. We also probed for tubulin-α and lamin B1 to monitor the distribution of the cytoskeleton and the nuclear envelope, respectively. Tubulin-α was highly enriched in the cytoplasmic fraction while lamin B1 was mainly localised in the nuclear fraction and a small portion seemed to be chromatin-bound. Having determined that the cellular compartments had been separated successfully, we analysed CA and IN protein levels in the different subcellular fractions by immunoblotting with anti-HIV-1 CA and anti-HIV-1 IN antibodies. The amount of CA in the cytoplasm remained relatively constant throughout the course of the infection (S3B and S3D Fig). Conversely, IN levels decreased markedly between 8 and 24 hpi, suggesting that IN protein has a shorter half-life than CA, but this was observed for both WT and A14C/E45C samples (S3B and S3D Fig). A similar pattern was seen in the membrane fraction (S3C and S3E Fig), except that there was noticeably more CA detected here following infection with A14C/E45C VLP compared to WT VLP. This may reflect a stronger association of A14C/E45C CA with the plasma membrane or organelle membranes that are both connected to microtubules which bind CA, or perhaps increased association with the nuclear membrane as this is continuous with the ER which is detected in this fraction. Alternatively, it might just reflect reduced uncoating for the hyper-stable mutants so that more CA is associated with each particle. Surprisingly, we detected both CA and IN in nuclear fractions as early as 30 minutes post infection (Figs 6C and S3F). Although the levels of IN were similar between WT and A14C/E45C at each time point, there was more A14C/E45C CA than WT CA present until 30 hpi, again confirming the increased stability of the mutant CA. This suggests that viral cores travel to the nucleus faster than originally thought, and before the peak of reverse transcription at 6 hpi (see Fig 4). Other groups have also recently reported this phenomenon [39,40,43,44,48]. To investigate whether the CA present in the nuclear fractions was still disulphide cross linked, we analysed the fractions under non-reducing conditions as in Fig 5A. Fig 6D shows that the majority of the mutant CA present was indeed oligomerised, mainly as hexamers, whereas the WT CA was monomeric. Adding β-ME to reduce the disulphide linkages resulted in a single monomeric band for the mutant CA (Fig 6D). Finally, we were able to detect low levels of A14C/E45C CA in the chromatin fractions as early as 30 minutes post infection (Figs 6E and S3G). However, the WT CA was undetectable at most time points (Figs 6E and S3G). Analysis of the CA in the chromatin fraction under non-reducing conditions revealed that, as in the nuclear fraction, most of the mutant CA was present as hexamers (Fig 6F). Unexpectedly, there was more IN present in the chromatin fraction from the WT infections than the A14C/E45C infections at all time points (Figs 6E and S3G). This suggests that although there was more CA present for the mutant, presumably because it was present as a more complete lattice, there was probably a lower number of individual particles associated with the chromatin than for WT infections, as indicated by the IN levels.

The association of A14C/E45C CA with nuclear fractions, particularly with the chromatin-bound fraction, suggests that, despite being hyper-stable, this CA protein could enter the nucleus. However, as the infected cells were dividing, it was possible that nuclear entry occurred during mitosis rather than through a NPC. To address this, we treated 293T or HeLa cells with aphidicolin to inhibit the cell cycle at G1/S and prevent mitosis. Arrested cells were then infected in the presence of aphidicolin and analysed for infectivity and 2-LTR circle production (S4A–S4C Fig). HeLa cells were also fractionated and nuclear and chromatin fractions analysed for the presence of CA and IN (S4D–S4F Fig). Although aphidicolin treatment slightly reduced the infectivity of both mutant and WT VLP (S4D Fig), the hyper-stable mutants were not affected more than WT (S4A and S4B Fig) and the ability of the mutants to form 2-LTR circles relative to WT was not impaired (S4C Fig). Furthermore, both CA and IN from A14C/E45C mutant VLP infections were detected in the nuclear and chromatin fractions of aphidicolin-treated cells at similar levels to the untreated cells (S4E and S4F Fig). This suggests that the presence of mutant CA in nuclear fractions was not simply due to nuclear entry during mitosis. Alternatively, as the nuclear envelope marker, lamin B1, was also distributed in these two fractions, it could be hypothesised that A14C/E45C was accumulating at the nuclear envelope rather than inside the nucleus. To help resolve this issue, we performed cellular localisation experiments.

### The A14C/E45C mutant is impeded at nuclear entry

**Fig 7:**
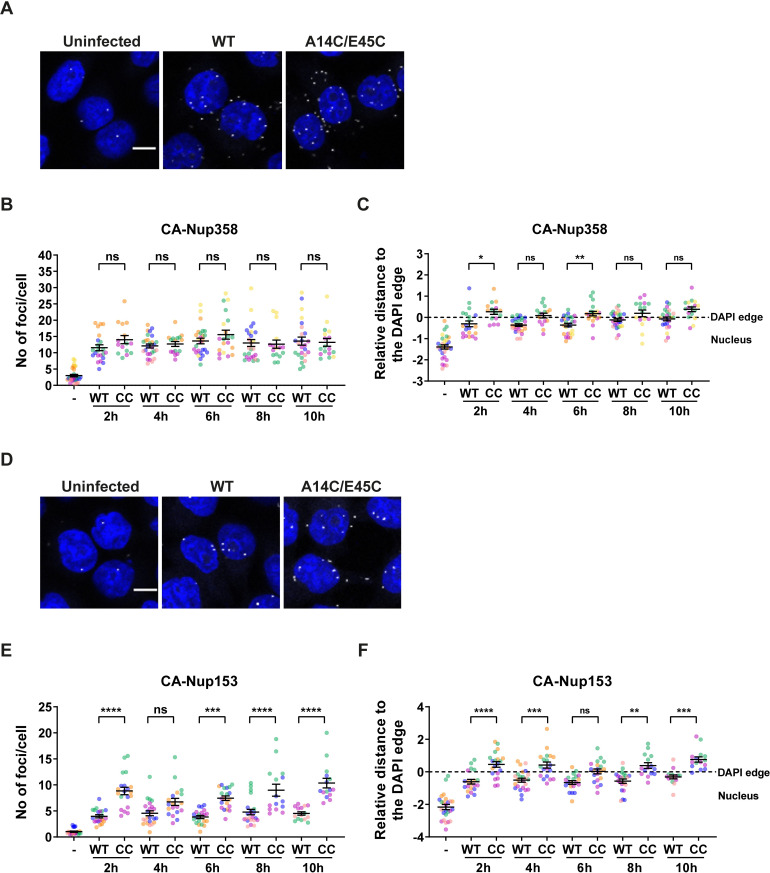
CA co-localisation of CA with NUP358 and NUP153 during infection. HeLa cells were synchronously infected with equal RT units of WT or A14C/E45C VLP and fixed at 2, 4, 6, 8 and 10 hpi. Cells were incubated with primary antibodies to HIV-1 CA and either NUP358 (A-C) or NUP153 (D-F), followed by specific secondary antibodies conjugated to the PLUS and MINUS PLA oligonucleotides (probes). Each foci represents a positive PLA signal generated by the amplification of the interaction between the PLUS and MINUS probes. (A) and (D) show representative images of CA-NUP358 and CA-NUP153 co-localisation at 8 hpi, respectively (the scale bar is 10μm). Longitudinal Z-series were acquired with a 63X objective using a confocal microscope followed by 3D image analysis performed with the GIANI plug-in in FIJI. The number of foci per cell (B and E) and the relative distance of those foci to the DAPI edge (C and F) were quantified. In the plots, each point represents the mean foci data from all the cells within an image (75 to 100 cells/image). At least 12 images were acquired per condition over at least three independent biological repeats (plotted in different colours). Overall means ± SEM are shown in black. * = p<0.05, ** = p<0.01, *** = p<0.001, **** = p<0.0001, ns = not significant.

In order to investigate nuclear entry of the A14C/E45C mutant further, we monitored the interactions between CA and the nuclear pore proteins NUP358 and NUP153 during infection. These proteins are part of the NPC where NUP358 faces the cytoplasm and NUP153 forms part of the nuclear basket of the NPC, facing the nucleus. Furthermore, both proteins have previously been described to bind HIV-1 CA directly and to be involved in HIV-1 replication [25,26,30]. HeLa cells were synchronously infected with equal RT units of WT or A14C/E45C VLP for 2, 4, 6, 8 and 10 hpi. At each time point, cells were fixed, and proximity ligation assays (PLA) were performed with specific antibody combinations: anti-HIV-1 CA and anti-NUP358 (Fig 7A–7C), and anti-HIV-1 CA and anti-NUP153 (Fig 7D–7F). In this assay, direct protein-protein interactions or proteins spaced < 40 nm apart can be visualised as foci by immunofluorescence. In addition, by doing *post hoc* analysis, the relative localisation of these interactions can be compared in relation to the nuclear envelope, in this case approximated by DAPI staining. Fig 7A shows representative cells assayed for CA-NUP358 co-localisation at 8 hpi. Counting the number of foci per cell (Fig 7B) revealed that there were similar levels of interaction between CA and NUP358 throughout the time course of infection, and there was no significant difference between WT and A14C/E45C infections. The localisation of these foci (Fig 7C) seemed to be similarly around the DAPI edge for both VLP, but with a tendency for WT foci to be further into the nucleus than A14C/E45C foci. This suggests that there is little difference in the early stages of infection, up to reaching the nuclear pore, between WT and A14C/E45C VLP, and, once again, that cores reach the nucleus within 2 hpi, sooner than previously expected. In contrast, there were clear differences in the staining for CA-NUP153 co-localisation between WT and A14C/E45C infections (Fig 7D–7F). Analysis of the number of foci per cell showed that there was increased A14C/E45C foci compared to WT infections at all time points (Fig 7E). As equal numbers of cores were arriving at the nucleus, as measured by CA-NUP358 staining (Fig 7B), this suggests that the A14C/E45C cores were spending more time at the nuclear pore. Moreover, comparing the localisation of the VLP showed that the A14C/E45C foci appeared to localise more towards the cytoplasmic side of the DAPI edge while the WT foci were mainly on the nuclear side (Fig 7F). Given that a layer of lamin and the nuclear envelope surround the chromatin in the nucleus, the DAPI edge does not indicate the exact nuclear/cytoplasmic boundary, but rather the inner side of the nuclear pore. Thus, being on the cytoplasmic side of the DAPI staining, together with increased CA-NUP153 staining, suggests that A14C/E45C hyper-stable cores are being trapped at the NPC.

### CPSF6 is not re-localised to nuclear speckles during infection with hyper-stable mutants

**Fig 8:**
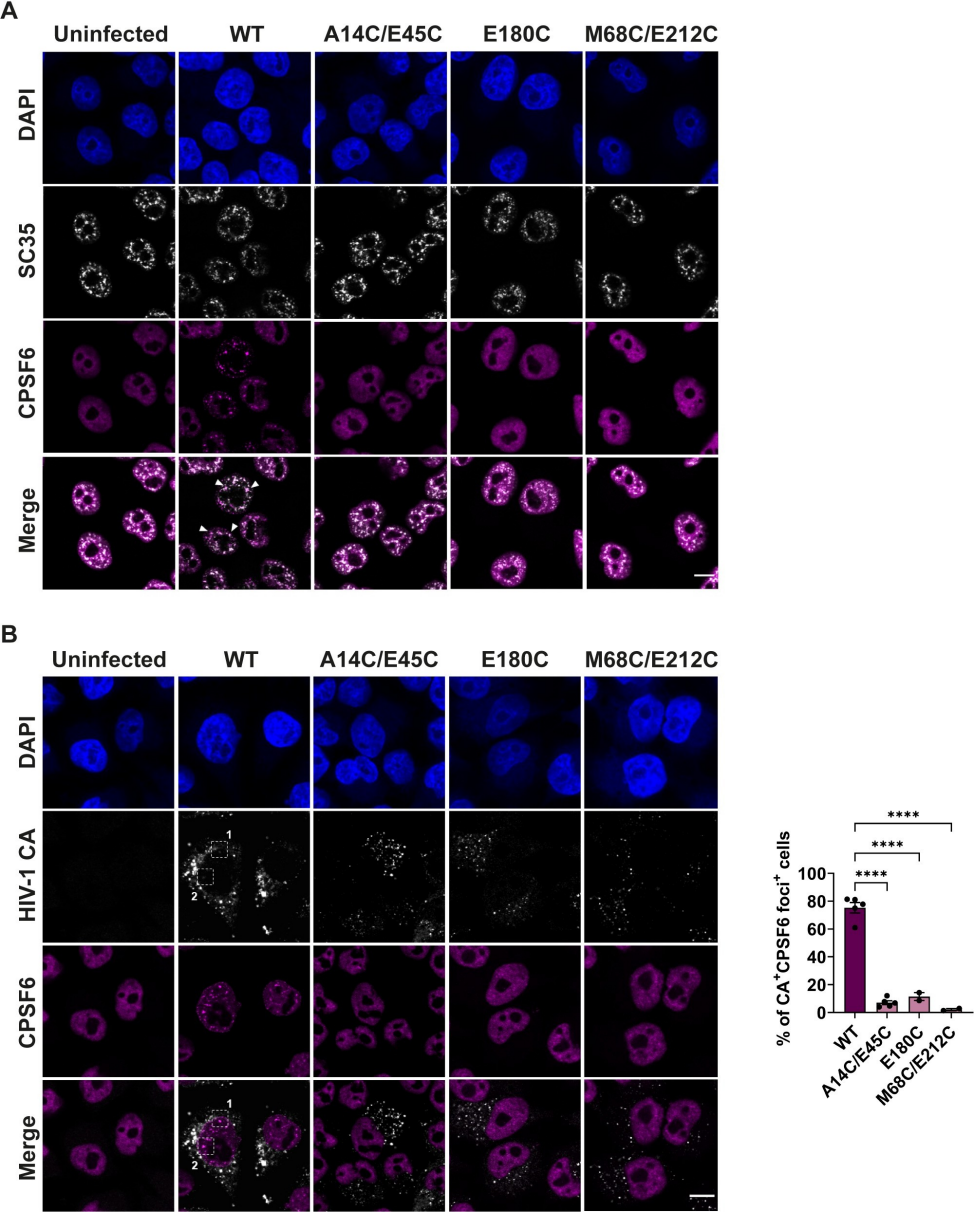
HIV-1 CA, CPSF6 and SC35 staining during infection with WT and hyper-stable mutant VLP. HeLa cells were synchronously infected with equal RT units of WT, A14C/E45C, E180C or M68C/E212C VLP and fixed at 16hpi. Cells were incubated with primary antibodies against HIV-1 CA, CPSF6 and SC35 followed by specific secondary antibodies conjugated to Alexa Fluor fluorophores. The scale bars are 10μm. (A) Representative images of SC35 and CPSF6 staining. White arrows point to SC35 and CPSF6 co-localisation in the merged WT image. (B) Representative images of HIV-1 CA and CPSF6 staining. White dashed line boxes in WT columns indicate zoomed-in regions labelled 1 and 2 (shown in S5A Fig). Bar chart shows the number of CA positive cells that showed CPSF6 redistribution to puncta. Points indicate individual biological repeats (~150 cells per experiment were used for quantification) and lines show the mean ± SEM; **** = p<0.0001.

In addition to binding nuclear pore proteins, CA interacts with CPSF6, which has been reported to direct HIV-1 integration site specificity. Specifically, CPSF6 is proposed to direct HIV-1 to highly transcribing regions of the genome identified as nuclear speckle-associated domains (SPADs) [9,34,35]. The absence of CPSF6, or blocking of CPSF6-CA binding, promotes integration into lamina-associated domains (LADs), regions of heterochromatin near the nuclear envelope [9,34]. Unfortunately, despite repeated attempts, we were unable to optimise our PLA assay to reliably visualise CA-CPSF6 co-localisation. However, recently, it has been observed that CPSF6 only redistributes to nuclear speckles during WT HIV-1 infection and not during infection with CPSF6-binding deficient mutants A77V and N74D [35,48]. This suggests that the CA-CPSF6 interaction is driving CPSF6 reorganisation in the nucleus. Therefore, to investigate the interaction between our hyper-stable mutants and CPSF6, we monitored the reorganisation and redistribution of CPSF6 to nuclear speckles during infection. HeLa cells were synchronously infected with equal RT units of WT, A14C/E45C, E180C or M68C/E212C VLP. At 16 hpi, cells were fixed and immuno-stained with antibodies against HIV-1 CA, CPSF6 and SC35, also called serine and arginine rich splicing factor 2 (SRSF2), which is a marker for nuclear speckles [64]. Due to the species of the antibodies, we could only co-stain for pairs of markers at a time. Figs 8A and S5B show that cells infected with WT-HIV-1 exhibit a compelling redistribution of CPSF6 into puncta that co-localise with SC35-positive nuclear speckles, confirming previous reports [35,48]. However, there was no such redistribution of CPSF6 during infection with any of the hyper-stable mutants which showed similar CPSF6 staining to uninfected cells. Importantly, when we co-stained for CA and CPSF6, ~80% of WT CA positive cells contained CPSF6 puncta (Fig 8B), compared to only 5% of hyper-stable CA positive cells. Although we were able to detect some CA signal in the nucleus of WT HIV-1 infected cells that colocalised with the CPSF6 signal by immunofluorescence (S5A Fig), the CA signal was relatively weak and we cannot say whether this CA was part of a core or not. The CA staining in the nucleus was not as evident during infection with the hyper-stable mutants. Together, these data show that WT CA is able to interact with CPSF6 and induce its redistribution to nuclear speckles, whilst the hyper-stable CA fail to alter CPSF6 localisation.

## Discussion

It has been known for many years that HIV-1 can infect non-dividing cells and so must cross the nuclear envelope during infection. Until recently, the dogma was that HIV-1 cores uncoated in the cytoplasm during reverse transcription, because they were too large to cross the nuclear pore. However, new evidence has led people to re-examine this order of events. When and where the CA lattice breaks down is currently highly controversial. Theories range from cytoplasmic uncoating to uncoating at the nuclear pore (reviewed in [65]) [35,38,39,42,66], and more recently, uncoating inside the nucleus [43,44,48,62]. Along these lines, emerging electron microscopy images appear to show intact cores going through nuclear pores and the size of the pore itself has been re-evaluated [62]. Some open questions are (1) Can reverse transcription complete inside a core? (2) Can a complete core cross the nuclear pore? (3) What replication events require CA? and (4) When is uncoating required? Unfortunately, uncoating is difficult to measure directly. Therefore, in this study, we have taken a genetic approach to investigating uncoating by comparing the replication of WT HIV-1 with hyper-stable CA mutants that are potentially slower or unable to uncoat. Thus, we have not asked “where/when does uncoating occur?”, but rather “where/when does viral replication get blocked if uncoating is prevented?”.

We compared a panel of mutants designed to induce disulphide bonds at different CA lattice interfaces. Many of these have previously been used for structural studies and clearly induced stability of *in vitro* assembled CA without altering overall CA structure [7,8,11,12,57,67–70], but anecdotally were thought unlikely to make stabilising disulphide bonds in the reducing environment of the cytoplasm. Nevertheless, we found that two previously described intra-hexamer mutants, A14C/E45C and M68C/E212C [11,12,57], and a novel inter-hexamer mutant, E180C, all showed increased lattice stability in cells (Figs 2A, 2C and 6) and maintained disulphide bonds following infection (Figs 5A, 6D and 6F). Interestingly, these three mutants represent stabilising different interfaces within the CA lattice. Thus, it appears that no particular interface is dominant in lattice stability. It was not surprising that most of the mutants did not increase core stability, as there is a lot of variation in the exact Cβ-Cβ distances between individual residues, depending on the region of the lattice that they reside (i.e. depending on the curvature of that part of the fullerene cone [5,67]). This is especially true around the trimeric interface. It is intriguing that the NTD-NTD mutant A14C/E45C resulted in hyper-stable cores whilst the NTD-NTD mutant A42C/T54C did not, as they have near identical hexamer crystal structures [12,68]. However, the geometry and exact chemistry of the surrounding environment likely affect whether disulphide bonds form in cells. Although any mutations in CA have the potential to cause multiple effects on function, by using mutants that had previously been structurally characterised, and testing a panel of different mutants, we hoped to minimise this limitation and provide an alternative genetic approach to complement other techniques that have been used to study uncoating and nuclear entry.

### Hyper-stable mutants can reverse transcribe

As expected, although they produced normal viral titres as measured by particle release (S1 Fig), most of the mutants in the panel had reduced infectivity in a range of cell lines (Fig 3). The intra-hexamer mutants were the least infectious, perhaps highlighting the importance of residues within the CA N-terminal domain for early replication steps. The severity of the infectivity defect did not correlate with core stability, as measured by the fate-of-CA assay (Fig 2), but did generally reflect the level of reverse transcription (Fig 4G and 4H). The exceptions to this were the three mutants identified as hyper-stable. Surprisingly, despite uncoating previously being linked to reverse transcription [38,49,51,61,71], A14C/E45C and E180C were able to reverse transcribe close to WT levels in 293T, HeLa and cycling U937 cells and, furthermore, showed only a partial reduction in 2-LTR circle production in these cells (Figs 4 and 5). Formation of 2-LTR circles shows that reverse transcription can complete in a hyper-stable core and implies that it is not necessary for the core to fully uncoat in order to accommodate the double stranded DNA. These results are in line with previous studies showing that hyper-stable mutants E45A, Q63A/Q67A, 5Mut (Q67H/K70R/H87P/T107N/L111) and A14C/E45C can reverse transcribe [48,52,72,73] and with a recent report saying that reverse transcription can complete in whole WT cores [43,74]. However, it is possible that the hyper-stable cores are partially opened. Indeed, the production of 2-LTR circles would imply that the core was open to some extent in order for the cellular ligases to access the viral DNA. It has also been reported that the hyper-stable E45A CA mutant can still partially open [75]. Variability in the efficiency of disulphide bond formation within the bulk population means that some viral cores will likely be more stable than others and it may be that it is the less stablised virions that are able to form 2-LTR circles. Recent *in vitro* studies have suggested that reverse transcription increases the pressure inside the core [61], inducing mechanical changes in the capsid that progressively remodel the lattice [76,77] and may result in viral DNA loops bursting out of partially uncoated cores [78]. This agrees with our previous assessment that uncoating is triggered after first strand transfer during reverse transcription [51]. By inducing disulphide bond formation, we may have prevented some or all of these remodelling changes occurring in the hyper-stable mutant cores, and therefore prevented more extensive uncoating from occurring. This implies that whilst reverse transcription promotes uncoating, it is not dependent on the disassembly of the lattice. Instead, the virus appears to be using reverse transcription to time uncoating. Nonetheless, hyper-stability can be detrimental for reverse transcription as the M68C/E212C mutant showed a ~10-fold decrease in reverse transcription products compared to WT in all the cells lines studied (Figs 4D and 5). The cysteine residues introduced in M68C/E212C are located on the intra-hexamer NTD-CTD interface, which is important for the curvature of the lattice. Christensen *et al*. recently reported that the compound GS-CA1, which binds at the NTD-CTD interface, affected capsid integrity and strongly inhibited reverse transcription *in vitro*, and proposed that affecting the NTD-CTD interface introduces lattice strain and promotes capsid fracturing [78]. Thus, it could be possible that the formation of disulphide bonds at this specific interface affects the structure of the CA lattice in such a way that is not compatible with reverse transcription.

Importantly, even the mutants that reverse transcribed well were still markedly impaired for integration (Fig 5), presumably meaning that the cores are unable to open fully, or in the correct manner to allow integration. This agrees with various recent reports suggesting that a final capsid uncoating reaction needs to occur before integration can take place [35,39,42–44,48,62,66]. Stabilising the CA lattice by either mutation, or drugs, probably affects flexibility as well as lattice break down, so it is hard to separate whether the core needs to restructure in some way or to break apart completely. We therefore use the term “CA remodelling” to cover both of these events.

The link between reverse transcription and uncoating has recently taken a new twist, as the location of reverse transcription in the cell has been questioned. There is increasing evidence that CA-containing viral complexes reach the nucleus early, before reverse transcription has completed [35,43,44,48,79,80], and that reverse transcription actually completes in the nucleus [44]. In support of nuclear-associated reverse transcription, here, we also detected CA and, importantly, IN in nuclear fractions at 30 minutes post infection (Fig 6) and detected CA interacting with nuclear pore proteins at 2 hpi (Fig 7), well before the peak of reverse transcription at 6hpi (Fig 4). Interestingly, 2-LTR circle formation was inhibited in differentiated U937 cells for all the hyper-stable mutants (Fig 5E), suggesting a more severe block to nuclear entry in these cells. Indeed, completion of reverse transcription after nuclear entry could explain the reduction of reverse transcription product accumulation and the subsequent lack of formation of 2-LTR circles for the hyper-stable mutants in these cells.

### Hyper-stable CA mutants are compromised for nuclear entry

As two of our hyper-stable mutants seemed to have a major defect after reverse transcription, we asked whether these hyper-stable cores could enter the nucleus. 2-LTR circles are considered a surrogate for nuclear entry [81,82], and as A14C/E45C and E180C produced 2-LTR circles in 293T cells, HeLa cells and cycling U937 cells, albeit with a 5–10 fold decrease compared to WT (Fig 5), it suggested that at least a proportion of particles could enter the nucleus. Our subcellular fractionation experiments confirmed that both WT and hyper-stable CA could be detected in all fractions following infection, including both soluble nuclear and chromatin-associated fractions (Figs 6 and S3). To confirm where the nuclear envelope fractionated, we blotted for lamin B1 which is located inside the inner layer of the nuclear envelope. Lamin B1 was detected in the nucleus and, to a lesser extent, in the chromatin fraction (Fig 6B), suggesting that the CA in these fractions could be at the nuclear envelope as well as inside the nucleus. Importantly, chromatin is known to be tightly linked with nuclear pores and lamin [83]. In the two nuclear fractions, there was noticeably less WT HIV-1 CA present at each time point than the hyper-stable mutant CA, suggesting that it was turned over faster (Figs 6C, 6E, S3F and S3G), although it is also possible that WT CA was more susceptible to degradation during the fractionation process. Conversely, the levels of IN were similar at all time points between WT and hyper-stable infections in all fractions except the chromatin-associated fraction, where there was more IN detected following WT infections. We assume that the IN levels represent the number of PICs and indicate that similar numbers of particles are present for WT and mutant until the chromatin fraction. The increased CA levels in each fraction following infection with the A14C/E45C mutant suggest that there is more CA present per particle, presumably reflecting the increase in core stability. As WT CA was barely detectable in the chromatin fraction (Fig 6E and S3G Fig), this could reflect WT cores being able to breakdown and deliver IN more efficiently to the chromatin than A14C/E45C cores which appear to retain some CA in the chromatin fraction (Figs 6E and S3G). Once disassembled, the CA is likely degraded. Together, these data show that although the hyper-stable cores retain more CA than WT cores, the IN levels are comparable until the cores meet chromatin. This implies that uncoating has little impact on replication until a chromatin associated event.

To explore whether hyper-stable cores could truly enter the nucleus or were associating with nuclear membranes, we studied the dynamics of WT and A14C/E45C CA interactions with nuclear pore proteins (Fig 7). We chose NUP358 and NUP153 because they are located on the outer and inner face of the nuclear pore, respectively, and because their interaction with CA is well characterised [25,26,29,30]. We found that WT and A14C/E45C CA showed similar levels of interaction with NUP358 over time (Fig 7B) suggesting that both cores could reach the nuclear pore with equivalent kinetics, agreeing with our fractionation experiments. As seen in a previous report [84], we observed some CA-NUP358 foci in the cytoplasm, which was more noticeable during A14C/E45C infection. Nuclear pore proteins are known to be dynamic, and co-localisation may occur away from the NPC [84], but there were no significant differences between the WT and mutant CA distribution (Fig 7C). In contrast, there was clear divergence in the co-localisation of CA and NUP153 between WT and hyper-stable cores. There were significantly increased numbers of foci for the A14C/E45C CA at all time points. As equal numbers of cores were arriving at the nucleus (as measured by NUP358 co-localisation), this suggests that the A14C/E45C cores were spending more time at the nuclear pore. Moreover, the A14C/E45C foci appeared to localise more towards the cytoplasmic side of the DAPI edge while the WT foci were mainly on the nuclear side, suggesting that WT cores travel further into the nucleus and that A14C/E45C hyper-stable cores are trapped at the NPC. It is worth pointing out that quantifying the distance of the PLA foci to the DAPI edge has caveats. The PLA signal is the result of a complex of rolling circle amplification products together with antibodies bound to two proteins of interest. As this is a big complex, the foci distance to the DAPI edge cannot be taken as an absolute distance. Furthermore, these observations could also be explained by invaginations of the nuclear membrane or limited resolution in the Z-axis that make PLA puncta at the top or bottom of the NE (in Z) appear to be in the nucleus. However, the same caveats apply to both WT and mutant cores, so a relative comparison can be made. Thus, it seems that uncoating or lattice flexibility is required for nuclear entry and points to a CA remodelling event at the nuclear pore, as suggested by some other labs [42,62]. It is interesting to recall that the A14C/E45C mutant still produces 2-LTR circles, despite apparent limited nuclear entry, questioning what this product really represents.

Interestingly, studies with the CPSF6 binding-defective HIV-1 CA mutants, N74D and A77V, have reported that a longer residence time at the nuclear envelope promotes integration into heterochromatin regions close to the nuclear envelope [9,34,35,43,48]. Furthermore, unlike WT HIV-1, neither the N74D nor the A77V mutant can re-localise CPSF6 to nuclear speckles, showing that this is a CA dependent event [9,34,35,48]. Since A14C/E45C cores were stalled at the nuclear pore, we were intrigued to see whether this mutant would be able to promote CPSF6 redistribution to nuclear speckles. In agreement with very recent reports [35,48], we found that only WT-infected cells showed a redistribution of CPSF6 to SC35 positive puncta (Fig 8). Importantly, A14C/E45C is still able to bind CPSF6 *in vitro* [85,86], suggesting that either it does not have access to CPSF6 in cells, or that it is unable to relocate to nuclear speckles because it is retained elsewhere. CPSF6 binds to the same pocket on the CA lattice as NUP153 [29,31,32] and Bejarano *et al* have recently reported the consecutive binding of the hexameric CA lattice to NUP153 and then CPSF6 in macrophages [40]. Thus, we speculate that if A14C/E45C is still binding NUP153 at the nuclear pore, it might not be able to uncouple and move on to binding CPSF6. We hypothesize that the CA lattice needs to remodel at the pore in order to be released from NUP153 and move into the nucleus, where it can then interact with CPSF6 and move to an optimal site for integration. As CA is required for the CPSF6 interaction, it follows that a fraction of CA must be retained by the pre-integration complex until chromatin binding, but sufficient CA must be removed to allow IN and the viral cDNA to access chromatin for integration itself to occur.

### Models of HIV-1 uncoating

**Fig 9:**
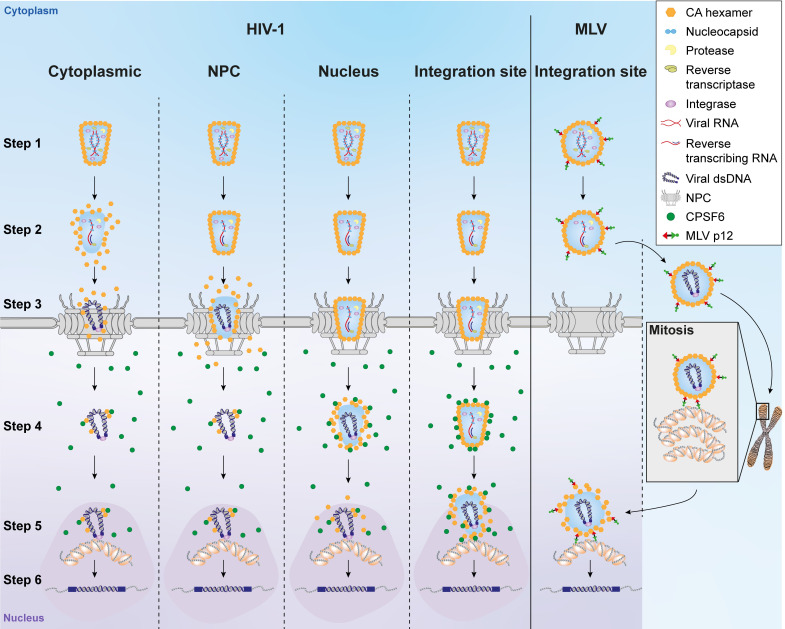
Current models for HIV-1 and MLV uncoating. Incoming viral cores (HIV-1, left columns and MLV, right column) surrounded by a CA lattice (orange hexagons) contain the viral RNA (red) coated with nucleocapsid (light blue), as well as the viral enzymes, protease (yellow), reverse transcriptase (light green) and integrase (purple) (step 1). Reverse transcription of the RNA to double stranded DNA (dark blue) starts following infection and the core travels towards the nucleus down microtubules (step 2). HIV-1 DNA can cross the nuclear pore (step 3), and in association with CPSF6 (green dots) (step 4), move to nuclear speckles (darker pink shaded regions) and integrate into the host cell chromatin (grey with orange ovals) (step 5) to form a provirus (step 6). When and where the CA lattice disassembles and where reverse transcription finishes is still debated and the possible scenarios for HIV-1 uncoating (loss of orange hexagons) are illustrated here: from left to right; uncoating in the cytoplasm, uncoating at the nuclear pore, uncoating inside the nucleus or uncoating at the site of integration. In contrast, MLV cannot pass through nuclear pores and must wait for mitosis to occur before accessing the chromatin. Our current model for MLV uncoating is shown on the right: At mitosis, the N-terminal domain of the MLV p12 protein (red) binds directly to CA and the C-terminal domain (green) binds to nucleosomes, tethering the likely intact MLV core to the host chromatin. Following mitosis, MLV uncoats to allow integration, which, as it is already associated with chromatin, probably occurs at the integration site.

We have illustrated the current models of HIV-1 uncoating in Fig 9. From left to right, uncoating in the cytoplasm, uncoating at the nuclear pore, uncoating inside the nucleus or uncoating at the integration site. Early uncoating or a failure to uncoat both have detrimental effects on virus infectivity. The data presented here point to a remodelling event at the nuclear envelope that, although it is not required for reverse transcription to complete, is needed to completely pass through the nuclear pore, allow binding to CPSF6 and, ultimately, for integration. We therefore think it likely that some sort of uncoating event begins at the nuclear pore and finishes at the site of integration. We have also previously shown that the murine leukaemia virus (MLV) p12 protein binds directly to both CA and nucleosomes, tethering the MLV core to chromatin during mitosis, and have proposed that p12 could be acting in a similar capacity to CPSF6 in HIV-1 infection [87]. Fig 9 includes our model of MLV uncoating. As MLV cannot traverse nuclear pores [88] it is unlikely that it undergoes a similar pore-induced CA remodelling event, suggesting that MLV uncoating might be triggered by chromatin binding. As MLV must wait for mitosis, it is possible that an intact CA lattice is needed until integration to provide a protective environment for the viral cDNA.

In conclusion, we have demonstrated that HIV-1 with a hyper-stable CA lattice is able to reverse transcribe successfully but is stalled at nuclear entry, which has a negative effect on CPSF6 binding and integration. We suggest that an uncoating or CA remodelling event normally occurs at the nuclear pore and that this is essential for the later replication events that take place in the nucleus. Furthermore, our observations suggest that viral cores are present at the nucleus before reverse transcription is completed. Therefore, it is plausible that both reverse transcription and uncoating finish in the nucleus. Further work is needed to fully understand the state of the CA lattice in the nucleus and the exact relationship between uncoating and reverse transcription.

## Material & methods

### Cell lines

Adherent cell lines, 293T and HeLa cells, were maintained in Dulbecco’s modified Eagle medium (Thermo Fisher), and suspension cell lines, SupT1 and U937 cells, were maintained in RPMI-1640 (Thermo Fisher). All cell lines were authenticated and tested mycoplasma-free from Bishop laboratory cell stocks. Media was supplemented with 10% heat-inactivated foetal bovine serum (FBS; Biosera) and 1% Penicillin/Streptomycin (Sigma). Cells were grown in a humidified incubator at 37°C and 5% CO_2_. Cycling U937 cells were differentiated by culturing the cells in 200nM Phorbol 12-myristate 13-acetate (PMA; Sigma) for 5 days. PMA was maintained in the media at a 100nM throughout the experiments.

### Plasmids and site-directed mutagenesis

The plasmids used to produce HIV-1 VLP, pVSV-G, pCMVΔR8.91, pCSGW and pWPTS-nlsLacZ, have been described previously [89,90]. To create Gag-Pol plasmids carrying cysteine-substitution mutations in CA, site-directed mutagenesis was performed on pCMVΔR8.91 using the QuickChange II-XL site-directed mutagenesis kit (Agilent) according to manufacturer’s instructions and using the primers listed in S1 Table. Repeated site-directed mutagenesis was performed to create the double, triple and quadruple mutants. The introduction of the desired mutations was confirmed by Sanger sequencing (Source Bioscience).

### Virus-like particle (VLP) production

HIV-1 virus-like particles (VLP) were produced by co-transfecting 293T cells with a 1:1:1 ratio of three plasmids: pVSV-G, pCSGW (GFP-reporter) or pWPTS-nlsLacZ (LacZ-reporter) and pCMVΔ8.91 (or pCMVΔ8.91 mutants). Approximately 16h post-transfection, cells were treated with 10mM sodium butyrate for 8h and VLP-containing supernatants were harvested 24h later. VLP titres were analysed using a Lenti RT ELISA kit (Cavidi) following manufacturer’s instructions. For the fate-of-capsid, cell fractionation and PLA assays, VLPs were concentrated by ultracentrifugation through a 20% (w/w) sucrose cushion using a Beckman SW32Ti rotor (Beckman-Coulter) at 20,200 rpm for 2h at 4°C.

### Single round infectivity assay

293T, HeLa, SupT1 and U937 cells (both cycling and differentiated) were challenged with normalised amounts of WT and mutant VLP based on their RT activity and incubated for 72 h at 37°C. The percentage of GFP-expressing cells was analysed by flow cytometry using a FACS VERSE, LSR or Fortessa A analysers (BD Biosciences). Data was analysed using FlowJo software. Cells infected with LacZ-expressing VLP were lysed in Tropix Lysis buffer (Life technologies) and frozen at -20°C. Cell lysates were mixed with Tropix galactostar reaction mixture (Life technologies) and luminescence was measured for 1h at 10min intervals on a Tecan Safire plate reader.

### Immunoblotting

Cells were lysed in ice-cold radioimmunoprecipitation assay (RIPA) buffer (Thermo Fisher) supplemented with a protease inhibitor cocktail (Roche). To assess disulphide cross-linking in cells, 2x non-reducing Laemmli SDS sample buffer (in house stock) was added to cell lysates or pelleted VLP (centrifuged at maximum speed in a bench top mini-centrifuge for 1h and at 4°C) prior to treatment with 50mM iodoacetamide (Sigma) or 10% β-Mercaptoethanol (Sigma). Samples treated with iodoacetamide were incubated for 30min at room temperature followed by a 15 min incubation at 65°C. Samples treated with β-Mercaptoethanol were incubated for 15min at room temperature and then boiled at 95°C for 5 min. Samples were then applied to 4–12% NUPAGE Bis-Tris gels (Thermo Fisher) and electrophoresed in MES SDS running buffer (Thermo Fisher). Primary antibodies used were: anti-HIV-1 CA (in house), anti-HIV-1 IN (in house), anti-HSP90 (CST; #4874), anti-calnexin (CST; #2679), anti-HDAC2 (CST; #5113), anti-histone 3 (CST; #14269), anti-tubulin-α (Bio-Rad; VMA00051) and anti-lamin B1 (Proteintech; 66095-1-Ig). Secondary antibodies used were: anti-mouse and anti-rabbit HRP-conjugated secondary antibodies (Thermo Fisher; 61–6520 and 31460); anti-mouse IRDye 800CW secondary antibodies (LICOR). Blots were analysed on a Chemidoc MP imaging system (Bio-Rad) or by X-ray film exposure or on an Odyssey CLx imaging system (LICOR).

### Fate-of-capsid assay

The fate-of-capsid assay was performed as previously described [58] but with some modifications. HeLa cells were seeded at 10^6^ cells/well in 6-well plates one day prior infection and spinoculated (1600rpm at 16°C for 30min, followed by 37°C for 1h) with equal amounts of VLP based on their RT activity. Cells were harvested at 2 and 20 hpi and cell pellets were lysed by adding hypotonic buffer (10mM Tris-HCl pH 8.0, 10mM KCl and 1mM EDTA pH 8.0 supplemented with a protease inhibitor cocktail (Roche)) and passing through a Qiashredder column (Qiagen). At this point, 50 μL was harvested as the input (I) and resuspended in 2x Laemmli’s SDS-PAGE sample loading buffer (Sigma). The remaining lysate was layered on top of a 30% (w/w) sucrose cushion and centrifuged using a Beckman SW41 rotor (Beckman Coulter) at 32,000rpm and 4°C for 1h, to separate soluble and assembled CA. After centrifugation, 500 μL of the uppermost portion of the supernatant was harvested as the soluble fraction (S). Following aspiration of the sucrose cushion, the pellet (P) was resuspended in 100 μL of 2x Laemmli’s SDS-PAGE sample loading buffer (Sigma). The soluble fraction was precipitated using methanol-chloroform extraction and resuspended in 100 μL of 2x Laemmli’s SDS-PAGE sample loading buffer (Sigma). Then, the I, S and P samples were analysed by western blotting with an anti-HIV-1 CA antibody. The densitometry of the CA blots was performed using the gel analysis tool in FIJI.

### Quantitative PCR analysis to measure RT products

Quantitative PCR analysis was conducted as previously described [51,91]. Briefly, VLP were treated with 20 units/mL RQ1-DNase (Promega) in 10mM MgCl_2_ for 1h at 37°C before infection. 293T, HeLa or U937 cells were spinoculated (1600rpm at 16°C for 30min) followed by a 30min incubation at 37°C. Cells were harvested at the indicated times post-infection and total DNA was extracted using the DNeasy Blood & Tissue kit (Qiagen). The extracted DNA was digested with 1 unit/μL DpnI (Thermo Fisher) for 2.5h at 37°C. qPCR was performed in TaqMan real-time PCR master mix (Thermo Fisher) with 900 nM primers and 250 nM probes. The reactions were performed on a 7500 fast real-time PCR system (Applied Biosystems). To calculate DNA copy numbers, standard curves were generated from serial dilutions of pCSGW or p2-LTR junction in 293T, HeLa or U937 cellular DNA as appropriate. The following primers and probes were used; strong stop cDNA products: *for* 5’-*TAACTAGGGAACCCACTGC*, *rev* 5’-*GCTAGAGATTTTCCACACTG* and *probe* 5’-*FAM-ACACAACAGACGGGCACACACTA-TAMRA*; second strand cDNA products: *for* 5’-*TAACTAGGGAACCCACTGC*, *rev* 5’-*CTGCGTCGAGAGAGCTCCTCTGGTT* and *probe* 5’-*FAM-ACACAACAGACGGGCACACACTA-TAMRA*; 2-LTR junction: *for* 5’-*GTGTGTGCCCGTCTGTTG*, *rev* 5’-*CAGTACAAGCAAAAAGCAGATC* and *probe* 5’-*FAM-GGTAACTAGAGATCCCTCAGACC-TAMRA*.

### Cell fractionation assay

HeLa cells were infected with equal amounts of WT and mutant VLP (based on their RT activity) by spinoculation (1600rpm, 16°C for 2h) followed by a 30 min incubation at 37°C. At the indicated times post-infection, cells were harvested in parallel either as a whole cell lysate (WCL) or for processing with the subcellular protein fractionation kit for cultured cells (Thermo Fisher) following manufacturer’s instructions. Protein content in the WCL and in the different fractions was measured by BCA assay (Thermo Fisher) using a FLUOstar Omega plate reader (BMG Labtech). Relative amounts of each fraction compared to the WCL were analysed by immunoblotting. The densitometry of the CA and IN bands was performed using the gel analysis tool in FIJI.

### Cell cycle arrest

Cell cycle arrest at the G1/S boundary was induced using 2μg/ml aphidicolin in DMSO. HeLa or 293T cells were treated with DMSO or aphidicolin for 24h prior infection and treatment was maintained throughout the experiments.

### Immunofluorescence

HeLa cells were seeded at 0.8x10^5^ cells/well on 13 mm glass coverslips in 24-well plates the day prior to infection. Cells were infected with equal RT units of WT or mutant VLP by spinoculation (1600rpm, 16°C for 2h) followed by a 30min incubation at 37°C. At the indicated times post-infection, cells were washed twice with ice-cold PBS, fixed with PBS supplemented with 4% paraformaldehyde for 5min at room temperature followed by an ice-cold methanol incubation for 5min at -20°C, and washed twice again with ice-cold PBS. After permeabilization with 0.5% saponin (Sigma) in PBS for 30min at room temperature, cells were blocked in 5% donkey serum (DS; Sigma) and 0.5% saponin in PBS for, at least, 1h at room temperature. Then, cells were incubated with the following primary antibodies: anti-HIV-1 CA (in house), anti-CPSF6 (Atlas; HPA039973) and anti-SC35 (Abcam; ab11826), diluted in 1% DS with 0.5% saponin in PBS (antibody buffer) for 1h at RT. After three washes with PBS, cells were incubated with the following secondary antibodies: goat anti-mouse-AF488 (Abcam; ab150117), donkey anti-rabbit-AF568 (Abcam; ab175692), donkey anti-mouse-AF647 (Thermo Fisher; A-31571) and donkey anti-rabbit-AF647 (Thermo Fisher; A-31573) in antibody buffer for 1h at RT. After three washes with PBS, the coverslips were mounted on glass slides with ProLong gold antifade mountant with DAPI (Thermo Fisher).

### Proximity ligation assay (PLA)

HeLa cells were seeded at 10^5^ cells/well on 13mm glass coverslips in 24-well plates the day prior to infection. Cells were infected with equal RT units of WT or mutant VLP by spinoculation (1600rpm, 16°C for 2h) followed by a 30min incubation at 37°C. At the indicated times post-infection, cells were washed twice with ice-cold PBS, fixed with PBS supplemented with 4% paraformaldehyde for 5min at room temperature followed by an ice-cold methanol incubation for 5min at -20°C, and washed twice again with ice-cold PBS. Then the immunofluorescence protocol was followed using the following pairs of primary antibodies: anti-HIV-1 CA (in house) and anti-NUP358 (Abcam; ab64276); anti-HIV-1 CA (in house) and anti-NUP153 (Abcam; ab84872), diluted in 1% DS with 0.5% saponin in PBS for 1h at RT. From this point, the Duolink PLA fluorescence detection kit’s protocol (Sigma) was followed. After three washes with PBS, coverslips were incubated with secondary antibodies conjugated to PLA probes (anti-mouse PLUS or anti-rabbit MINUS; Sigma) in antibody buffer (Sigma) for 1h at 37°C. Coverslips were incubated with ligase for 30 min at 37°C followed by amplification by polymerase for 100 min at 37°C. From the primary antibody incubation, coverslips were washed with buffers A and B (Sigma) as indicated in the manufacturer’s protocol. Finally, coverslips were mounted with the Duolink *in situ* mounting media with DAPI (Sigma) on glass slides (Menzel-Gläser) and sealed. Samples were visualised on a SP5 inverted confocal microscope (Leica) using a 63X 1.3NA oil immersion objective (Leica). Longitudinal Z-series were acquired with 0.5 μm step sizes.

Image analysis was performed using the GIANI plug-in in Fiji (described in [92] and in https://github.com/djpbarry/Giani/wiki). The number of foci and their distance to the edge of the DAPI staining, were quantified taking into account the 3D cell volume reflected by the longitudinal Z-series. In brief, the nuclei were first detected using the advanced nuclear blob detector in the DAPI channel where the seed points are the centre of each nuclei. The advanced detector uses a Determinant-of-Hessian approach to blob detection, based on the calculation of Hessian eigenimages implemented by ImageScience. Following filtering, the nuclei were segmented using the Li threshold and the nuclear volume marker option, where boundaries between nuclei are created based solely on distance from seed points. Each resulting boundary line is equidistant from the seed points in the nuclei that it bounds. This is implemented using Thomas Boudier’s watershed segmentation. Then, the segmented nuclei were used as seed for segmenting the cells. After applying a Gaussian filter, the Huang threshold and the cell volume marker option were used to complete segmentation. Consequently, the PLA foci were measured using the “localise spots” option and the simple blob detector in the PLA signal channel. The blob radius (μm) and the threshold of detection were set up accurately by confirming that only PLA foci were detected through the Z-stack. After all the measurements were set up, the software automatically analysed all the images from the same experiment. The software determined the number of foci per cell and whether the foci were outside or inside the nucleus. It also measured the distance of these foci to the DAPI edge. Thus, if the distance value was negative the PLA spot was inside the DAPI edge and if it was positive, it was outside. From the output data, the number of foci and the relative distance to the DAPI edge were plotted.

### Statistics

Statistical analyses were carried out using GraphPad Prism 9 software. Differences between conditions was estimated by one-way ANOVA complemented with Turkey’s *post hoc* test (*, P < 0.05; **, P < 0.01; ***, P < 0.001; ****, P < 0.0001).

## Supporting information

S1 Table Primers used to make CA mutations by site-directed mutagenesis. Table listing forward and reverse primers used to introduce cysteine mutations on CA by site-directed mutagenesis.
(DOCX)

S1 Fig VLP production and Gag expression. (A) GFP-reporter gene-expressing HIV-1 WT and CA mutant VLP were produced by transient transfection of 293T cells and the VLP titres in the cell supernatants were calculated by measuring RT activity using a modified RT ELISA. The bar chart shows the RT activity of the mutants relative to WT. Points indicate individual biological repeats and bars show the mean ± SEM. Colour coding is as in Fig 1. (B) Immunoblot of transfected 293T producer cell lysates probed with an anti-HIV-1 CA antibody showing expression of WT and mutant Gag proteins from CA mutants A14C/E45C, W184A/M185A and A14C/E45C/W184A/M185A. The blot was imaged using a LiCor Odyssey CLx imager. (C) Immunoblot of transfected 293T producer cell lysates probed with anti-HIV-1 CA antibody showing expression of WT and mutant Gag proteins from CA mutants A42C/T54C, Q63C/Y169C, E180C, V181C, L151C/L169C and K203C/A217C. The blot was imaged by exposure to X-ray film.
(TIF)

S2 Fig Effect of CA mutations on late reverse transcription. 293T cells were synchronously infected with equivalent RT units of WT or mutant VLP. Cells were harvested and DNA extracted and analysed for viral late cDNA products (second strand) by qPCR. (A) Bar chart shows the levels of second strand cDNA at 6 h post infection relative to WT infection. (B) Bar chart shows the levels of second strand cDNA at 24 h (left y-axis) and infectivity at 72 h (right y-axis) compared to WT VLP for each mutant. Individual points represent biological repeats and bars indicate the mean ± SEM. (C) Bar chart shows the ratio of relative levels of second strand cDNA to infectivity, from (B). Dashed line indicates a ratio of 3. Bars are colour coded according to the lattice interface at which the cysteines have been introduced, as in Fig 1. Hyper-stable mutants are indicated with black arrow heads.
(TIF)

S3 Fig Localisation and quantification of CA and IN proteins in subcellular fractions during WT and A14C/E45C infections. HeLa cells were synchronously infected with equal RT units of WT or A14C/E45C mutant (CC in the figure) VLP. At either 0, 0.5 and 2 hpi, or 4, 8, 24 and 30 hpi, cells were harvested in parallel to be processed as a whole cell lysate or to undergo subcellular fractionation. Protein levels were quantified by BCA assay, proportional amounts of the fractions related to the WCL were loaded on SDS-PAGE gels and analysed by immunoblotting using the following antibodies: Anti-CA and anti-IN for HIV-1 proteins, anti-HSP90 for cytoplasm, anti-calnexin for membranes, anti-HDAC2 for nucleus and anti-histone 3 for chromatin. (B, C) Panels show representative immunoblots probed for HIV-1 CA and IN and the appropriate fractionation marker: (B) Cytoplasm fraction with HSP90 as a loading control, (C) membrane fraction with calnexin as a loading control. “-”indicates uninfected cells. (A, D, E, F, G) Bar charts show the densitometry analysis of the immunoblots plotted as the ratio of CA or IN proteins to the loading control. (A) whole cell lysates, (D) cytoplasm, (E) membranes, (F) nucleus and (G) chromatin fractions. Bar charts show mean ± SEM of at least two independent repeats. The key for bar chart colour coding is shown at the top of figure.
(TIF)

S4 Fig Effect of Aphidicolin in WT and hyper-stable VLP infection. 293T or HeLa cells were treated with either DMSO or aphidicolin at 2μg/ml for 24h, in order to arrest the cells in the G1/S boundary, prior to synchronous infection with WT or mutant VLP. Aphidicolin was maintained in the culture media throughout infection. (A) Treated 293T cells were infected with equal RT units of LacZ-reporter WT or mutant VLP. Cells were lysed at 36hpi and LacZ activity was measured using a chemilumescent assay. The data is shown as % of LacZ activity relative to WT VLP. (B) Treated HeLa cells were infected with equal RT units of GFP-reporter WT or mutant VLP. The percentage of GFP+ cells was measured by flow cytometry at 36hpi and plotted relative to WT VLP. (C) 293T cells were infected with equal RT units of LacZ-reporter WT or mutant VLP. At 24hpi, cells were harvested for DNA extraction and 2-LTR circles were measured by qPCR. All data are plotted relative to WT infections (shown as a dashed line at 100%). Points indicate individual biological repeats and lines show the mean ± SEM. (D-F) Treated HeLa cells were synchronously infected with equal RT units of WT or A14C/E45C mutant (CC) VLP. At 0 (harvested after spinoculation) and 4hpi, cells were harvested in parallel to be processed as a whole cell lysate (WCL) or to undergo subcellular fractionation as in Fig 6. Protein levels were quantified by BCA assay, proportional amounts of the fractions related to the WCL were loaded on SDS-PAGE gels and analysed by immunoblotting for HIV-1 CA or IN and a fractionation marker. Panels show representative immunoblots from two independent experiments of (D) WCL with HSP90 as a loading control, (E) Nuclear fraction with HDAC2 as a loading control, (F) Chromatin fraction with histone3 as a loading control. The first lanes on each blot are uninfected cells (Neg). +/- indicates whether the cells were treated with aphidicolin or DMSO respectively prior to infection.
(TIF)

S5 Fig HIV-1 CA, CPSF6 and SC35 staining during infection. HeLa cells were synchronously infected with equal RT units of WT, A14C/E45C, E180C or M68C/E212C VLP and fixed at 16hpi. Cells were incubated with primary antibodies against HIV-1 CA and CPSF6 (A) or CPSF6 and SC35 (B) followed by specific secondary antibodies conjugated to Alexa Fluor fluorophores. (A) shows zoomed-in images of regions labelled 1 and 2 in Fig 8B. White arrows point to HIV-1 CA and CPSF6 co-localisation. The scale bar is 2 μm. (B) shows representative images of CPSF6 and SC35 staining at a lower magnification (63X) than in Fig 8A. The scale bar is 20 μm.
(TIF)

## References

[1] McDonald D, Vodicka MA, Lucero G, Svitkina TM, Borisy GG, Emerman M, et al. Visualization of the intracellular behavior of HIV in living cells. J Cell Biol. 2002;159(3):441–52. doi: 10.1083/jcb.200203150 12417576 PMC2173076

[2] Ganser BK, Li S, Klishko VY, Finch JT, Sundquist WI. Assembly and analysis of conical models for the HIV-1 core. Science. 1999;283(5398):80–3. doi: 10.1126/science.283.5398.80 9872746

[3] Ganser-Pornillos BK, von Schwedler UK, Stray KM, Aiken C, Sundquist WI. Assembly properties of the human immunodeficiency virus type 1 CA protein. J Virol. 2004;78(5):2545–52. doi: 10.1128/jvi.78.5.2545-2552.2004 14963157 PMC369201

[4] Pornillos O, Ganser-Pornillos BK, Yeager M. Atomic-level modelling of the HIV capsid. Nature. 2011;469(7330):424–7. doi: 10.1038/nature09640 21248851 PMC3075868

[5] Mattei S, Glass B, Hagen WJ, Krausslich HG, Briggs JA. The structure and flexibility of conical HIV-1 capsids determined within intact virions. Science. 2016;354(6318):1434–7. doi: 10.1126/science.aah4972 27980210

[6] Du S, Betts L, Yang R, Shi H, Concel J, Ahn J, et al. Structure of the HIV-1 full-length capsid protein in a conformationally trapped unassembled state induced by small-molecule binding. J Mol Biol. 2011;406(3):371–86. doi: 10.1016/j.jmb.2010.11.027 21146540 PMC3194004

[7] Deshmukh L, Schwieters CD, Grishaev A, Ghirlando R, Baber JL, Clore GM. Structure and dynamics of full-length HIV-1 capsid protein in solution. J Am Chem Soc. 2013;135(43):16133–47. doi: 10.1021/ja406246z 24066695 PMC3946434

[8] Zhao G, Perilla JR, Yufenyuy EL, Meng X, Chen B, Ning J, et al. Mature HIV-1 capsid structure by cryo-electron microscopy and all-atom molecular dynamics. Nature. 2013;497(7451):643–6. doi: 10.1038/nature12162 23719463 PMC3729984

[9] Li W, Singh PK, Sowd GA, Bedwell GJ, Jang S, Achuthan V, et al. CPSF6-Dependent Targeting of Speckle-Associated Domains Distinguishes Primate from Nonprimate Lentiviral Integration. mBio. 2020;11(5). doi: 10.1128/mBio.02254-20 32994325 PMC7527728

[10] Briggs JA, Wilk T, Welker R, Krausslich HG, Fuller SD. Structural organization of authentic, mature HIV-1 virions and cores. EMBO J. 2003;22(7):1707–15. doi: 10.1093/emboj/cdg143 12660176 PMC152888

[11] Pornillos O, Ganser-Pornillos BK, Kelly BN, Hua Y, Whitby FG, Stout CD, et al. X-ray structures of the hexameric building block of the HIV capsid. Cell. 2009;137(7):1282–92. doi: 10.1016/j.cell.2009.04.063 19523676 PMC2840706

[12] Pornillos O, Ganser-Pornillos BK, Banumathi S, Hua Y, Yeager M. Disulfide bond stabilization of the hexameric capsomer of human immunodeficiency virus. J Mol Biol. 2010;401(5):985–95. doi: 10.1016/j.jmb.2010.06.042 20600115 PMC3050670

[13] Ganser-Pornillos BK, Yeager M, Pornillos O. Assembly and architecture of HIV. Adv Exp Med Biol. 2012;726:441–65. doi: 10.1007/978-1-4614-0980-9_20 22297526 PMC6743068

[14] Gres AT, Kirby KA, KewalRamani VN, Tanner JJ, Pornillos O, Sarafianos SG. STRUCTURAL VIROLOGY. X-ray crystal structures of native HIV-1 capsid protein reveal conformational variability. Science. 2015;349(6243):99–103. doi: 10.1126/science.aaa5936 26044298 PMC4584149

[15] Le Sage V, Mouland AJ, Valiente-Echeverria F. Roles of HIV-1 capsid in viral replication and immune evasion. Virus Res. 2014;193:116–29. doi: 10.1016/j.virusres.2014.07.010 25036886

[16] Rasaiyaah J, Tan CP, Fletcher AJ, Price AJ, Blondeau C, Hilditch L, et al. HIV-1 evades innate immune recognition through specific cofactor recruitment. Nature. 2013;503(7476):402–5. doi: 10.1038/nature12769 24196705 PMC3928559

[17] Stremlau M, Owens CM, Perron MJ, Kiessling M, Autissier P, Sodroski J. The cytoplasmic body component TRIM5alpha restricts HIV-1 infection in Old World monkeys. Nature. 2004;427(6977):848–53. doi: 10.1038/nature02343 14985764

[18] Malim MH, Bieniasz PD. HIV Restriction Factors and Mechanisms of Evasion. Cold Spring Harb Perspect Med. 2012;2(5):a006940. doi: 10.1101/cshperspect.a006940 22553496 PMC3331687

[19] Franke EK, Yuan HE, Luban J. Specific incorporation of cyclophilin A into HIV-1 virions. Nature. 1994;372(6504):359–62. doi: 10.1038/372359a0 7969494

[20] Bosco DA, Eisenmesser EZ, Pochapsky S, Sundquist WI, Kern D. Catalysis of cis/trans isomerization in native HIV-1 capsid by human cyclophilin A. Proc Natl Acad Sci U S A. 2002;99(8):5247–52. doi: 10.1073/pnas.082100499 11929983 PMC122755

[21] Gamble TR, Vajdos FF, Yoo S, Worthylake DK, Houseweart M, Sundquist WI, et al. Crystal structure of human cyclophilin A bound to the amino-terminal domain of HIV-1 capsid. Cell. 1996;87(7):1285–94. doi: 10.1016/s0092-8674(00)81823-1 8980234

[22] Sabo Y, Walsh D, Barry DS, Tinaztepe S, de Los Santos K, Goff SP, et al. HIV-1 induces the formation of stable microtubules to enhance early infection. Cell Host Microbe. 2013;14(5):535–46. doi: 10.1016/j.chom.2013.10.012 24237699 PMC3855456

[23] Lukic Z, Dharan A, Fricke T, Diaz-Griffero F, Campbell EM. HIV-1 uncoating is facilitated by dynein and kinesin 1. J Virol. 2014;88(23):13613–25. doi: 10.1128/JVI.02219-14 25231297 PMC4248982

[24] Malikov V, da Silva ES, Jovasevic V, Bennett G, de Souza Aranha Vieira DA, Schulte B, et al. HIV-1 capsids bind and exploit the kinesin-1 adaptor FEZ1 for inward movement to the nucleus. Nat Commun. 2015;6:6660. doi: 10.1038/ncomms7660 25818806 PMC4380233

[25] Brass AL, Dykxhoorn DM, Benita Y, Yan N, Engelman A, Xavier RJ, et al. Identification of host proteins required for HIV infection through a functional genomic screen. Science. 2008;319(5865):921–6. doi: 10.1126/science.1152725 18187620

[26] Konig R, Zhou Y, Elleder D, Diamond TL, Bonamy GM, Irelan JT, et al. Global analysis of host-pathogen interactions that regulate early-stage HIV-1 replication. Cell. 2008;135(1):49–60. doi: 10.1016/j.cell.2008.07.032 18854154 PMC2628946

[27] Christ F, Thys W, De Rijck J, Gijsbers R, Albanese A, Arosio D, et al. Transportin-SR2 imports HIV into the nucleus. Curr Biol. 2008;18(16):1192–202. doi: 10.1016/j.cub.2008.07.079 18722123

[28] Fernandez J, Machado AK, Lyonnais S, Chamontin C, Gartner K, Leger T, et al. Transportin-1 binds to the HIV-1 capsid via a nuclear localization signal and triggers uncoating. Nat Microbiol. 2019;4(11):1840–50. doi: 10.1038/s41564-019-0575-6 31611641

[29] Matreyek KA, Engelman A. The requirement for nucleoporin NUP153 during human immunodeficiency virus type 1 infection is determined by the viral capsid. J Virol. 2011;85(15):7818–27. doi: 10.1128/JVI.00325-11 21593146 PMC3147902

[30] Di Nunzio F, Danckaert A, Fricke T, Perez P, Fernandez J, Perret E, et al. Human nucleoporins promote HIV-1 docking at the nuclear pore, nuclear import and integration. PLoS One. 2012;7(9):e46037. doi: 10.1371/journal.pone.0046037 23049930 PMC3457934

[31] Price AJ, Jacques DA, McEwan WA, Fletcher AJ, Essig S, Chin JW, et al. Host cofactors and pharmacologic ligands share an essential interface in HIV-1 capsid that is lost upon disassembly. PLoS Pathog. 2014;10(10):e1004459. doi: 10.1371/journal.ppat.1004459 25356722 PMC4214760

[32] Bhattacharya A, Alam SL, Fricke T, Zadrozny K, Sedzicki J, Taylor AB, et al. Structural basis of HIV-1 capsid recognition by PF74 and CPSF6. Proc Natl Acad Sci U S A. 2014;111(52):18625–30. doi: 10.1073/pnas.1419945112 25518861 PMC4284599

[33] Chin CR, Perreira JM, Savidis G, Portmann JM, Aker AM, Feeley EM, et al. Direct Visualization of HIV-1 Replication Intermediates Shows that Capsid and CPSF6 Modulate HIV-1 Intra-nuclear Invasion and Integration. Cell Rep. 2015;13(8):1717–31. doi: 10.1016/j.celrep.2015.10.036 26586435 PMC5026322

[34] Achuthan V, Perreira JM, Sowd GA, Puray-Chavez M, McDougall WM, Paulucci-Holthauzen A, et al. Capsid-CPSF6 Interaction Licenses Nuclear HIV-1 Trafficking to Sites of Viral DNA Integration. Cell Host Microbe. 2018;24(3):392–404 e8. doi: 10.1016/j.chom.2018.08.002 30173955 PMC6368089

[35] Francis AC, Marin M, Singh PK, Achuthan V, Prellberg MJ, Palermino-Rowland K, et al. HIV-1 replication complexes accumulate in nuclear speckles and integrate into speckle-associated genomic domains. Nat Commun. 2020;11(1):3505. doi: 10.1038/s41467-020-17256-8 32665593 PMC7360574

[36] Miller MD, Farnet CM, Bushman FD. Human immunodeficiency virus type 1 preintegration complexes: studies of organization and composition. J Virol. 1997;71(7):5382–90. doi: 10.1128/JVI.71.7.5382-5390.1997 9188609 PMC191777

[37] Fassati A, Goff SP. Characterization of intracellular reverse transcription complexes of human immunodeficiency virus type 1. J Virol. 2001;75(8):3626–35. doi: 10.1128/JVI.75.8.3626-3635.2001 11264352 PMC114854

[38] Mamede JI, Cianci GC, Anderson MR, Hope TJ. Early cytoplasmic uncoating is associated with infectivity of HIV-1. Proc Natl Acad Sci U S A. 2017;114(34):E7169–E78. doi: 10.1073/pnas.1706245114 28784755 PMC5576815

[39] Francis AC, Melikyan GB. Single HIV-1 Imaging Reveals Progression of Infection through CA-Dependent Steps of Docking at the Nuclear Pore, Uncoating, and Nuclear Transport. Cell Host Microbe. 2018;23(4):536–48 e6. doi: 10.1016/j.chom.2018.03.009 29649444 PMC5901770

[40] Bejarano DA, Peng K, Laketa V, Borner K, Jost KL, Lucic B, et al. HIV-1 nuclear import in macrophages is regulated by CPSF6-capsid interactions at the nuclear pore complex. Elife. 2019;8. 10.7554/eLife.41800 PMC6400501 30672737

[41] Zurnic Bonisch I, Dirix L, Lemmens V, Borrenberghs D, De Wit F, Vernaillen F, et al. Capsid-Labelled HIV To Investigate the Role of Capsid during Nuclear Import and Integration. J Virol. 2020;94(7). doi: 10.1128/JVI.01024-19 31941774 PMC7081887

[42] Blanco-Rodriguez G, Gazi A, Monel B, Frabetti S, Scoca V, Mueller F, et al. Remodeling of the Core Leads HIV-1 Preintegration Complex into the Nucleus of Human Lymphocytes. J Virol. 2020;94(11). doi: 10.1128/JVI.00135-20 32238582 PMC7269431

[43] Burdick RC, Li C, Munshi M, Rawson JMO, Nagashima K, Hu WS, et al. HIV-1 uncoats in the nucleus near sites of integration. Proc Natl Acad Sci U S A. 2020;117(10):5486–93. doi: 10.1073/pnas.1920631117 32094182 PMC7071919

[44] Dharan A, Bachmann N, Talley S, Zwikelmaier V, Campbell EM. Nuclear pore blockade reveals that HIV-1 completes reverse transcription and uncoating in the nucleus. Nat Microbiol. 2020;5(9):1088–95. doi: 10.1038/s41564-020-0735-8 32483230 PMC9286700

[45] Schaller T, Ocwieja KE, Rasaiyaah J, Price AJ, Brady TL, Roth SL, et al. HIV-1 capsid-cyclophilin interactions determine nuclear import pathway, integration targeting and replication efficiency. PLoS Pathog. 2011;7(12):e1002439. doi: 10.1371/journal.ppat.1002439 22174692 PMC3234246

[46] Hulme AE, Kelley Z, Foley D, Hope TJ. Complementary Assays Reveal a Low Level of CA Associated with Viral Complexes in the Nuclei of HIV-1-Infected Cells. J Virol. 2015;89(10):5350–61. doi: 10.1128/JVI.00476-15 25741002 PMC4442523

[47] Burdick RC, Delviks-Frankenberry KA, Chen J, Janaka SK, Sastri J, Hu WS, et al. Dynamics and regulation of nuclear import and nuclear movements of HIV-1 complexes. PLoS Pathog. 2017;13(8):e1006570. doi: 10.1371/journal.ppat.1006570 28827840 PMC5578721

[48] Selyutina A, Persaud M, Lee K, KewalRamani V, Diaz-Griffero F. Nuclear Import of the HIV-1 Core Precedes Reverse Transcription and Uncoating. Cell Rep. 2020;32(13):108201. doi: 10.1016/j.celrep.2020.108201 32997983 PMC7871456

[49] Hulme AE, Perez O, Hope TJ. Complementary assays reveal a relationship between HIV-1 uncoating and reverse transcription. Proc Natl Acad Sci U S A. 2011;108(24):9975–80. doi: 10.1073/pnas.1014522108 21628558 PMC3116424

[50] Yang Y, Fricke T, Diaz-Griffero F. Inhibition of reverse transcriptase activity increases stability of the HIV-1 core. J Virol. 2013;87(1):683–7. doi: 10.1128/JVI.01228-12 23077298 PMC3536417

[51] Cosnefroy O, Murray PJ, Bishop KN. HIV-1 capsid uncoating initiates after the first strand transfer of reverse transcription. Retrovirology. 2016;13(1):58. doi: 10.1186/s12977-016-0292-7 27549239 PMC4994286

[52] Forshey BM, von Schwedler U, Sundquist WI, Aiken C. Formation of a human immunodeficiency virus type 1 core of optimal stability is crucial for viral replication. J Virol. 2002;76(11):5667–77. doi: 10.1128/jvi.76.11.5667-5677.2002 11991995 PMC137032

[53] von Schwedler UK, Stray KM, Garrus JE, Sundquist WI. Functional surfaces of the human immunodeficiency virus type 1 capsid protein. J Virol. 2003;77(9):5439–50. doi: 10.1128/jvi.77.9.5439-5450.2003 12692245 PMC153941

[54] Link JO, Rhee MS, Tse WC, Zheng J, Somoza JR, Rowe W, et al. Clinical targeting of HIV capsid protein with a long-acting small molecule. Nature. 2020;584(7822):614–8. doi: 10.1038/s41586-020-2443-1 32612233 PMC8188729

[55] Bester SM, Wei G, Zhao H, Adu-Ampratwum D, Iqbal N, Courouble VV, et al. Structural and mechanistic bases for a potent HIV-1 capsid inhibitor. Science. 2020;370(6514):360–4. doi: 10.1126/science.abb4808 33060363 PMC7831379

[56] Srinivasan N, Sowdhamini R, Ramakrishnan C, Balaram P. Conformations of disulfide bridges in proteins. Int J Pept Protein Res. 1990;36(2):147–55. doi: 10.1111/j.1399-3011.1990.tb00958.x 2272751

[57] Yufenyuy EL, Aiken C. The NTD-CTD intersubunit interface plays a critical role in assembly and stabilization of the HIV-1 capsid. Retrovirology. 2013;10:29. doi: 10.1186/1742-4690-10-29 23497318 PMC3623829

[58] Yang Y, Luban J, Diaz-Griffero F. The fate of HIV-1 capsid: a biochemical assay for HIV-1 uncoating. Methods Mol Biol. 2014;1087:29–36. doi: 10.1007/978-1-62703-670-2_3 24158811 PMC4317566

[59] Rietsch A, Beckwith J. The genetics of disulfide bond metabolism. Annu Rev Genet. 1998;32:163–84. doi: 10.1146/annurev.genet.32.1.163 9928478

[60] Sevier CS, Kaiser CA. Formation and transfer of disulphide bonds in living cells. Nat Rev Mol Cell Biol. 2002;3(11):836–47. doi: 10.1038/nrm954 12415301

[61] Rankovic S, Varadarajan J, Ramalho R, Aiken C, Rousso I. Reverse Transcription Mechanically Initiates HIV-1 Capsid Disassembly. J Virol. 2017;91(12). doi: 10.1128/JVI.00289-17 28381579 PMC5446659

[62] Zila V, Margiotta E, Turonova B, Muller TG, Zimmerli CE, Mattei S, et al. Cone-shaped HIV-1 capsids are transported through intact nuclear pores. Cell. 2021. doi: 10.1016/j.cell.2021.01.025 33571428 PMC7895898

[63] Guedán A, Caroe ER, Barr GCR, Bishop KN. The Role of Capsid in HIV-1 Nuclear Entry. Viruses. 2021;13(8):1425. doi: 10.3390/v13081425 34452291 PMC8402913

[64] Fei J, Jadaliha M, Harmon TS, Li ITS, Hua B, Hao Q, et al. Quantitative analysis of multilayer organization of proteins and RNA in nuclear speckles at super resolution. J Cell Sci. 2017;130(24):4180–92. doi: 10.1242/jcs.206854 29133588 PMC5769577

[65] Campbell EM, Hope TJ. HIV-1 capsid: the multifaceted key player in HIV-1 infection. Nat Rev Microbiol. 2015;13(8):471–83. doi: 10.1038/nrmicro3503 26179359 PMC4876022

[66] Zila V, Muller TG, Laketa V, Muller B, Krausslich HG. Analysis of CA Content and CPSF6 Dependence of Early HIV-1 Replication Complexes in SupT1-R5 Cells. mBio. 2019;10(6). doi: 10.1128/mBio.02501-19 31690677 PMC6831778

[67] Byeon IJ, Meng X, Jung J, Zhao G, Yang R, Ahn J, et al. Structural convergence between Cryo-EM and NMR reveals intersubunit interactions critical for HIV-1 capsid function. Cell. 2009;139(4):780–90. doi: 10.1016/j.cell.2009.10.010 19914170 PMC2782912

[68] Yeager M. Design of in vitro symmetric complexes and analysis by hybrid methods reveal mechanisms of HIV capsid assembly. J Mol Biol. 2011;410(4):534–52. doi: 10.1016/j.jmb.2011.04.073 21762799 PMC3166646

[69] Zhao G, Ke D, Vu T, Ahn J, Shah VB, Yang R, et al. Rhesus TRIM5alpha disrupts the HIV-1 capsid at the inter-hexamer interfaces. PLoS Pathog. 2011;7(3):e1002009. doi: 10.1371/journal.ppat.1002009 21455494 PMC3063768

[70] Ramalho R, Rankovic S, Zhou J, Aiken C, Rousso I. Analysis of the mechanical properties of wild type and hyperstable mutants of the HIV-1 capsid. Retrovirology. 2016;13:17. doi: 10.1186/s12977-016-0250-4 26979152 PMC4793510

[71] Francis AC, Marin M, Shi J, Aiken C, Melikyan GB. Time-Resolved Imaging of Single HIV-1 Uncoating In Vitro and in Living Cells. PLoS Pathog. 2016;12(6):e1005709. doi: 10.1371/journal.ppat.1005709 27322072 PMC4913920

[72] Shi J, Zhou J, Shah VB, Aiken C, Whitby K. Small-molecule inhibition of human immunodeficiency virus type 1 infection by virus capsid destabilization. J Virol. 2011;85(1):542–9. doi: 10.1128/JVI.01406-10 20962083 PMC3014163

[73] Yang R, Shi J, Byeon IJ, Ahn J, Sheehan JH, Meiler J, et al. Second-site suppressors of HIV-1 capsid mutations: restoration of intracellular activities without correction of intrinsic capsid stability defects. Retrovirology. 2012;9:30. doi: 10.1186/1742-4690-9-30 22515365 PMC3351742

[74] Li C, Burdick RC, Nagashima K, Hu WS, Pathak VK. HIV-1 cores retain their integrity until minutes before uncoating in the nucleus. Proc Natl Acad Sci U S A. 2021;118(10). doi: 10.1073/pnas.2019467118 33649225 PMC7958386

[75] Marquez CL, Lau D, Walsh J, Shah V, McGuinness C, Wong A, et al. Kinetics of HIV-1 capsid uncoating revealed by single-molecule analysis. Elife. 2018;7. doi: 10.7554/eLife.34772 29877795 PMC6039174

[76] Rankovic S, Deshpande A, Harel S, Aiken C, Rousso I. HIV-1 uncoating occurs via a series of rapid biomechanical changes in the core related to individual stages of reverse transcription. J Virol. 2021. 10.1128/JVI.00166-21 PMC8139671 33692202

[77] Rankovic S, Ramalho R, Aiken C, Rousso I. PF74 Reinforces the HIV-1 Capsid To Impair Reverse Transcription-Induced Uncoating. J Virol. 2018;92(20). doi: 10.1128/JVI.00845-18 30089694 PMC6158434

[78] Christensen DE, Ganser-Pornillos BK, Johnson JS, Pornillos O, Sundquist WI. Reconstitution and visualization of HIV-1 capsid-dependent replication and integration in vitro. Science. 2020;370(6513). doi: 10.1126/science.abc8420 33033190 PMC8022914

[79] Francis AC, Marin M, Prellberg MJ, Palermino-Rowland K, Melikyan GB. HIV-1 Uncoating and Nuclear Import Precede the Completion of Reverse Transcription in Cell Lines and in Primary Macrophages. Viruses. 2020;12(11). 10.3390/v12111234 PMC7693591 33143125

[80] Rensen E, Mueller F, Scoca V, Parmar JJ, Souque P, Zimmer C, et al. Clustering and reverse transcription of HIV-1 genomes in nuclear niches of macrophages. EMBO J. 2021;40(1):e105247. doi: 10.15252/embj.2020105247 33270250 PMC7780146

[81] Sharkey ME, Teo I, Greenough T, Sharova N, Luzuriaga K, Sullivan JL, et al. Persistence of episomal HIV-1 infection intermediates in patients on highly active anti-retroviral therapy. Nat Med. 2000;6(1):76–81. doi: 10.1038/71569 10613828 PMC9513718

[82] Butler SL, Hansen MS, Bushman FD. A quantitative assay for HIV DNA integration in vivo. Nat Med. 2001;7(5):631–4. doi: 10.1038/87979 11329067

[83] Kuhn TM, Capelson M. Nuclear Pore Proteins in Regulation of Chromatin State. Cells. 2019;8(11). doi: 10.3390/cells8111414 31717499 PMC6912232

[84] Dharan A, Talley S, Tripathi A, Mamede JI, Majetschak M, Hope TJ, et al. KIF5B and Nup358 Cooperatively Mediate the Nuclear Import of HIV-1 during Infection. PLoS Pathog. 2016;12(6):e1005700. doi: 10.1371/journal.ppat.1005700 27327622 PMC4915687

[85] Price AJ, Fletcher AJ, Schaller T, Elliott T, Lee K, KewalRamani VN, et al. CPSF6 defines a conserved capsid interface that modulates HIV-1 replication. PLoS Pathog. 2012;8(8):e1002896. doi: 10.1371/journal.ppat.1002896 22956906 PMC3431306

[86] Rasheedi S, Shun MC, Serrao E, Sowd GA, Qian J, Hao C, et al. The Cleavage and Polyadenylation Specificity Factor 6 (CPSF6) Subunit of the Capsid-recruited Pre-messenger RNA Cleavage Factor I (CFIm) Complex Mediates HIV-1 Integration into Genes. J Biol Chem. 2016;291(22):11809–19. doi: 10.1074/jbc.M116.721647 26994143 PMC4882448

[87] Wanaguru M, Barry DJ, Benton DJ, O’Reilly NJ, Bishop KN. Murine leukemia virus p12 tethers the capsid-containing pre-integration complex to chromatin by binding directly to host nucleosomes in mitosis. PLoS Pathog. 2018;14(6):e1007117. doi: 10.1371/journal.ppat.1007117 29906285 PMC6021111

[88] Miller DG, Adam MA, Miller AD. Gene transfer by retrovirus vectors occurs only in cells that are actively replicating at the time of infection. Mol Cell Biol. 1990;10(8):4239–42. doi: 10.1128/mcb.10.8.4239-4242.1990 2370865 PMC360961

[89] Zufferey R, Nagy D, Mandel RJ, Naldini L, Trono D. Multiply attenuated lentiviral vector achieves efficient gene delivery in vivo. Nat Biotechnol. 1997;15(9):871–5. doi: 10.1038/nbt0997-871 9306402

[90] Bainbridge JW, Stephens C, Parsley K, Demaison C, Halfyard A, Thrasher AJ, et al. In vivo gene transfer to the mouse eye using an HIV-based lentiviral vector; efficient long-term transduction of corneal endothelium and retinal pigment epithelium. Gene Ther. 2001;8(21):1665–8. doi: 10.1038/sj.gt.3301574 11895005

[91] Bishop KN, Verma M, Kim EY, Wolinsky SM, Malim MH. APOBEC3G inhibits elongation of HIV-1 reverse transcripts. PLoS Pathog. 2008;4(12):e1000231. doi: 10.1371/journal.ppat.1000231 19057663 PMC2584787

[92] Barry DJ, Gerri C, Bell DM, D’Antuono R, Niakan KK. GIANI: open-source software for automated analysis of 3D microscopy images. bioRxiv. 2020. 10.1242/jcs.259511 PMC9189431 35502739

