# Supplementary material for: HIV-1 requires capsid remodelling at the nuclear pore for nuclear entry and integration
Source: PLoS Pathog. 2021 Sep 20;17(9):e1009484. doi: 10.1371/journal.ppat.1009484 (PMC8483370; doi:10.1371/journal.ppat.1009484)
Supplement: S1 Table — Table listing forward and reverse primers used to introduce cysteine mutations on CA by site-directed mutagenesis. (DOCX) [file ppat.1009484.s001.docx]

**S1 Table. Primers used to make CA mutations by site-directed mutagenesis.**

| **Mutation** | **Primer** | **Sequence** |
| --- | --- | --- |
| **P38A** | Forward | 5’-*GGCTTTCAGCCCAGAAGTGATAGCCATGTTTTCAGCATTA* |
|  | Reverse | 5’-*TAATGCTGAAAACATGGCTATCACTTCTGGGCTGAAAGCC* |
| **A14C** | Forward | 5’-*GGGGCAAATGGTACATCAGTGCATATCACCTAGAACTTTA* |
|  | Reverse | 5’-*TAAAGTTCTAGGTGATATGCACTGATGTACCATTTGCCCC* |
| **E45C** | Forward | 5’-*CATGTTTTCAGCATTATCATGCGGAGCCACCCCACAAGAT* |
|  | Reverse | 5’-*ATCTTGTGGGGTGGCTCCGCATGATAATGCTGAAAACATG* |
| **A42C** | Forward | 5’-*GTGATACCCATGTTTTCATGCTTATCAGAAGGAGCCACCCC* |
|  | Reverse | 5’-*GGGGTGGCTCCTTCTGATAAGCATGAAAACATGGGTATCAC* |
| **T54C** | Forward | 5’-*CCACCCCACAAGATTTAAACTGCATGCTAAACACAGTGGGGGG* |
|  | Reverse | 5’-*CCCCCCACTGTGTTTAGCATGCAGTTTAAATCTTGTGGGGTGG* |
| **M68C** | Forward | 5’-*GGGACATCAAGCAGCCATGCAATGCTTAAAAGAGACCATCAATG* |
|  | Reverse | 5’-*CATTGATGGTCTCTTTTAAGCATTGCATGGCTGCTTGATGTCCC* |
| **E212C** | Forward | 5’-*GGGACCAGGAGCGACACTATGCGAAATGATGACAGCATGTCAGGG* |
|  | Reverse | 5’-*CCCTGACATGCTGTCATCATTTCGCATAGTGTCGCTCCTGGTCCC* |
| **Q63C** | Forward | 5’-*GCTAAACACAGTGGGGGGACATTGCGCAGCCATGCAAATGTTAAAAG* |
|  | Reverse | 5’-*CTTTTAACATTTGCATGGCTGCGCAATGTCCCCCCACTGTGTTTAGC* |
| **Y169C** | Forward | 5’-*GAGACTATGTAGACCGATTCTGTAAAACTCTAAGAGCCGAGC* |
|  | Reverse | 5’-*GCTCGGCTCTTAGAGTTTTACAGAATCGGTCTACATAGTCTC* |
| **E180C** | Forward | 5’-*GAGCCGAGCAAGCTTCACAATGCGTAAAAAATTGGATGACAGAAACC* |
|  | Reverse | 5’-*GGTTTCTGTCATCCAATTTTTTACGCATTGTGAAGCTTGCTCGGCTC* |
| **V181C** | Forward | 5’-*GCCGAGCAAGCTTCACAAGAGTGCAAAAATTGGATGACAGAAACC* |
|  | Reverse | 5’-*GGTTTCTGTCATCCAATTTTTGCACTCTTGTGAAGCTTGCTCGGC* |
| **L151C** | Forward | 5’-*GTATAGCCCTACCAGCATTTGCGACATAAGACAAGGACCAAAGG* |
|  | Reverse | 5’-*CCTTTGGTCCTTGTCTTATGTCGCAAATGCTGGTAGGGCTATAC* |
| **L189C** | Forward | 5’-*GGTAAAAAATTGGATGACAGAAACCTGCTTGGTCCAAAATGCGAACCC* |
|  | Reverse | 5’-*GGGTTCGCATTTTGGACCAAGCAGGTTTCTGTCATCCAATTTTTTACC* |
| **A204C** | Forward | 5’-*GACTATTTTAAAATGTTTGGGACCAGGAGCGACACTAGAA* |
|  | Reverse | 5’-*CCCAAACATTTTAAAATAGTCTTACAATCTGGGTTCGCAT* |
| **P207C** | Forward | 5’-*GGGATGTGGAGCGACACTAGAAGAAATGATGACAGCATGT* |
|  | Reverse | 5’-*CGCTCCACATCCCAATGCTTTTAAAATAGTCTTACAATCT* |
| **T216C** | Forward | 5’-*GATGTGTGCATGTCAGGGAGTGGGGGGACCCGGCCATAAAG* |
|  | Reverse | 5’-*CCCTGACATGCACACATCATTTCTTCTAGTGTCGCTCCTG* |
| **K203C** | Forward | 5’-*CCCAGATTGTAAGACTATTTTATGCGCATTGGGACCAGGAGCGACAC* |
|  | Reverse | 5’-*GTGTCGCTCCTGGTCCCAATGCGCATAAAATAGTCTTACAATCTGGG* |
| **A217C** | Forward | 5’-*CACTAGAAGAAATGATGACATGCTGTCAGGGAGTGGGGGGACC* |
|  | Reverse | 5’-*GGTCCCCCCACTCCCTGACAGCATGTCATCATTTCTTCTAGTG* |
| **W184A/**  **M185A** | Forward | 5’-*GCTTCACAAGAGGTAAAAAATGCGGCGACAGAAACCTTGTTGGTCC* |
|  | Reverse | 5’-*GGACCAACAAGGTTTCTGTCGCCGCATTTTTTACCTCTTGTGAAGC* |
