# Supplementary figures and images for: HIV-1 requires capsid remodelling at the nuclear pore for nuclear entry and integration
Source: PLoS Pathog. 2021 Sep 20;17(9):e1009484. doi: 10.1371/journal.ppat.1009484 (PMC8483370; doi:10.1371/journal.ppat.1009484)

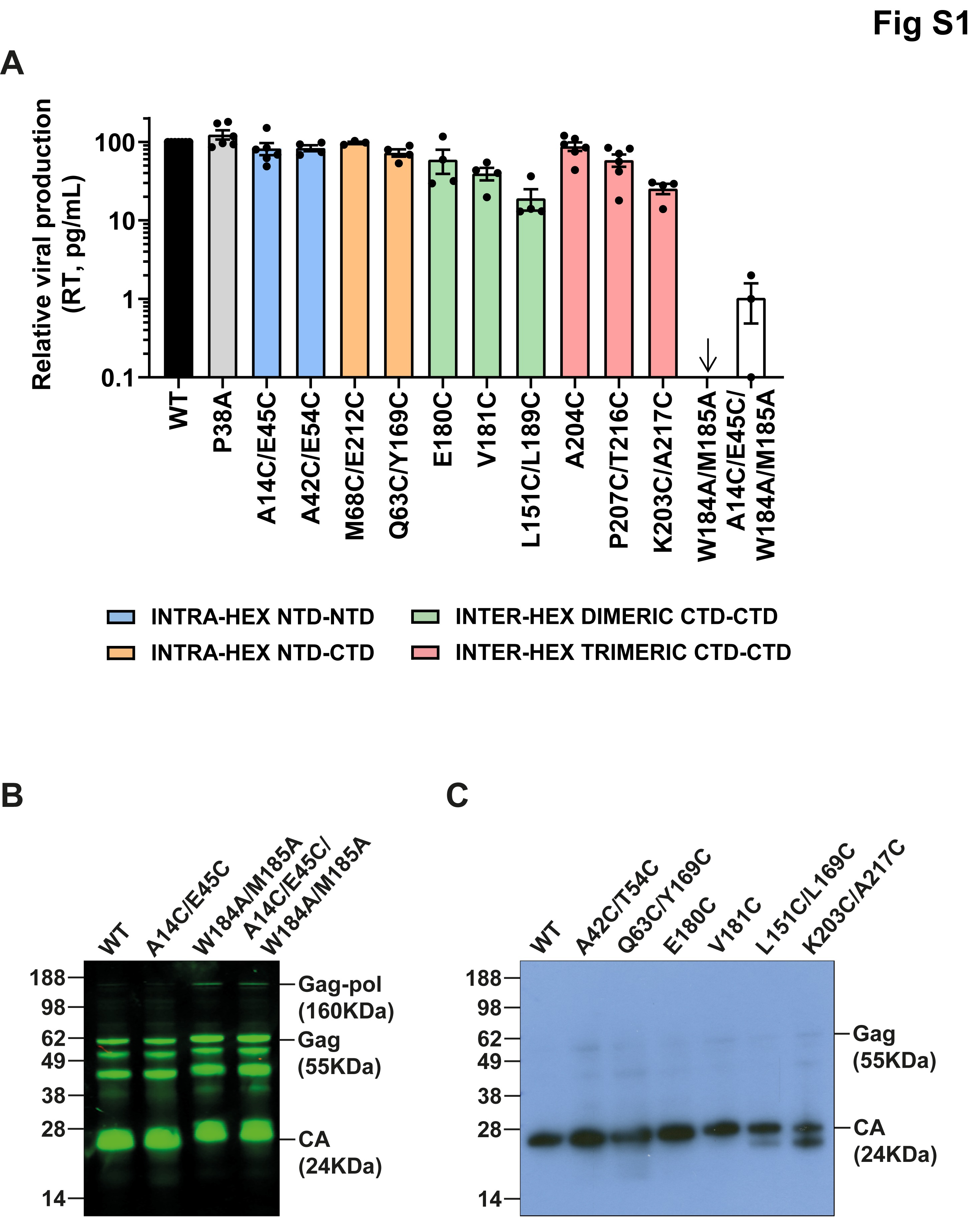

Supplement: S1 Fig — (A) GFP-reporter gene-expressing HIV-1 WT and CA mutant VLP were produced by transient transfection of 293T cells and the VLP titres in the cell supernatants were calculated by measuring RT activity using a modified RT ELISA. The bar chart shows the RT activity of the mutants relative to WT. Points indicate individual biological repeats and bars show the mean ± SEM. Colour coding is as in Fig 1. (B) Immunoblot of transfected 293T producer cell lysates probed with an anti-HIV-1 CA antibody showing expression of WT and mutant Gag proteins from CA mutants A14C/E45C, W184A/M185A and A14C/E45C/W184A/M185A. The blot was imaged using a LiCor Odyssey CLx imager. (C) Immunoblot of transfected 293T producer cell lysates probed with anti-HIV-1 CA antibody showing expression of WT and mutant Gag proteins from CA mutants A42C/T54C, Q63C/Y169C, E180C, V181C, L151C/L169C and K203C/A217C. The blot was imaged by exposure to X-ray film. (TIF) [file ppat.1009484.s002.tif]

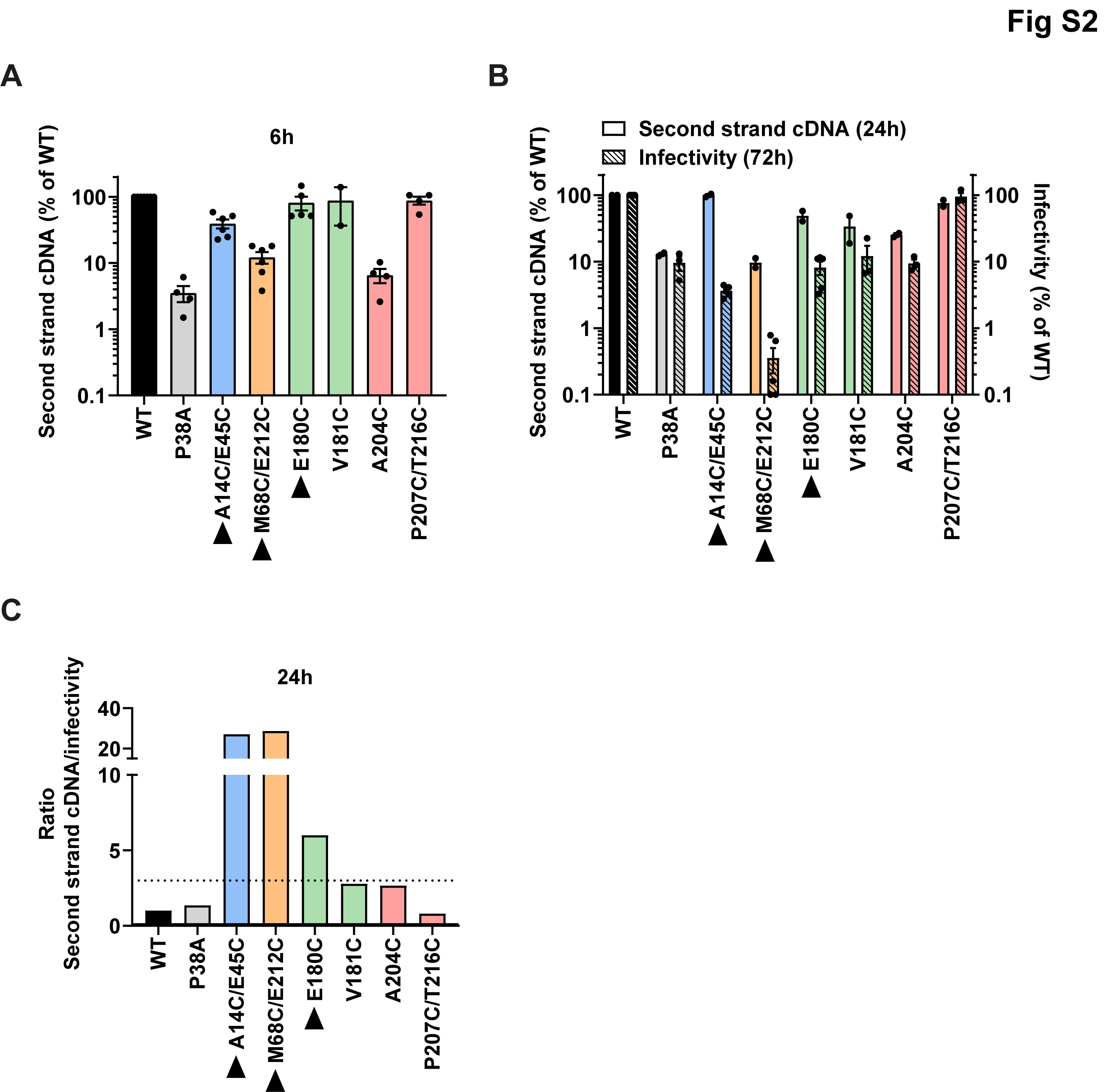

Supplement: S2 Fig — 293T cells were synchronously infected with equivalent RT units of WT or mutant VLP. Cells were harvested and DNA extracted and analysed for viral late cDNA products (second strand) by qPCR. (A) Bar chart shows the levels of second strand cDNA at 6 h post infection relative to WT infection. (B) Bar chart shows the levels of second strand cDNA at 24 h (left y-axis) and infectivity at 72 h (right y-axis) compared to WT VLP for each mutant. Individual points represent biological repeats and bars indicate the mean ± SEM. (C) Bar chart shows the ratio of relative levels of second strand cDNA to infectivity, from (B). Dashed line indicates a ratio of 3. Bars are colour coded according to the lattice interface at which the cysteines have been introduced, as in Fig 1. Hyper-stable mutants are indicated with black arrow heads. (TIF) [file ppat.1009484.s003.tif]

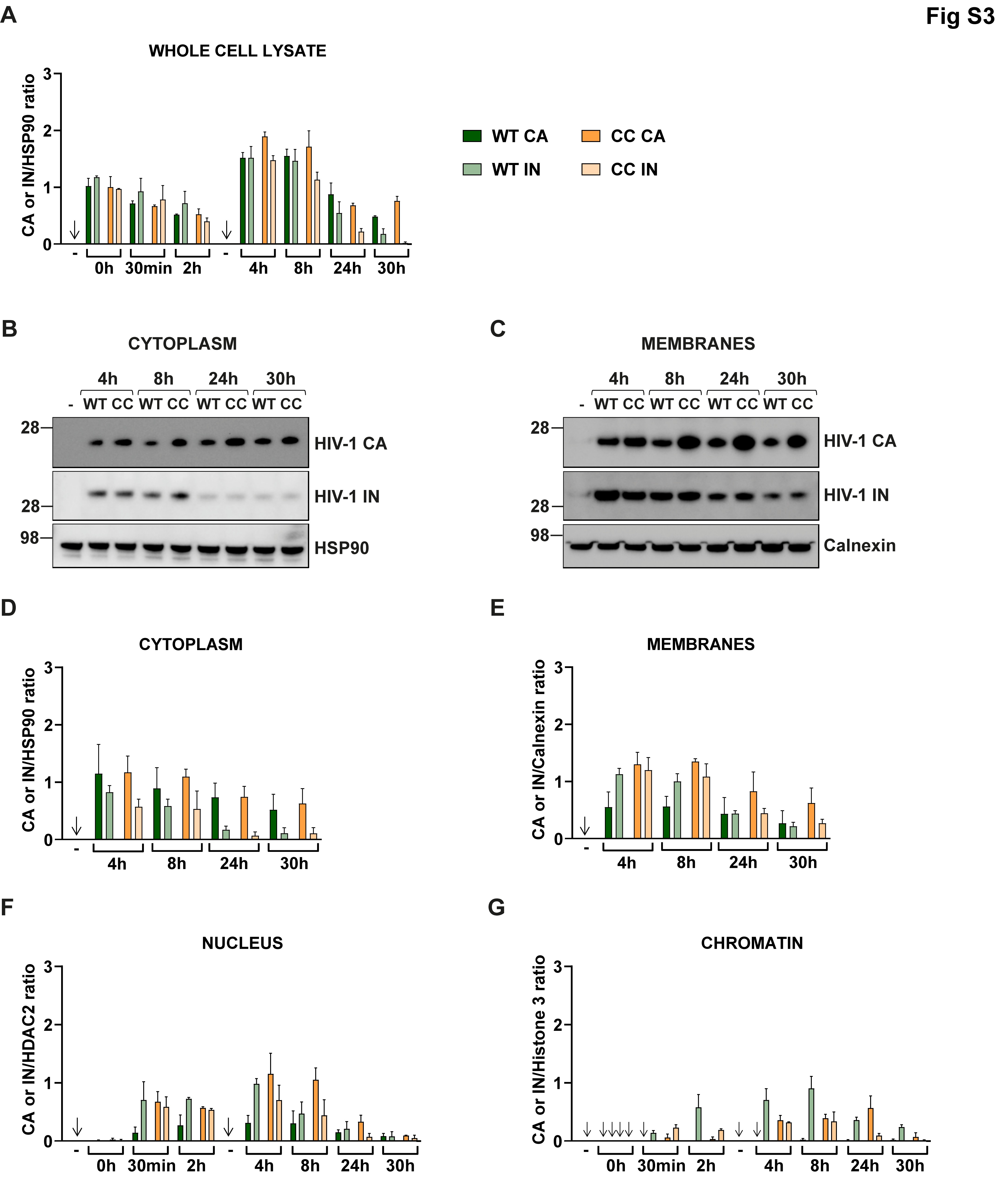

Supplement: S3 Fig — HeLa cells were synchronously infected with equal RT units of WT or A14C/E45C mutant (CC in the figure) VLP. At either 0, 0.5 and 2 hpi, or 4, 8, 24 and 30 hpi, cells were harvested in parallel to be processed as a whole cell lysate or to undergo subcellular fractionation. Protein levels were quantified by BCA assay, proportional amounts of the fractions related to the WCL were loaded on SDS-PAGE gels and analysed by immunoblotting using the following antibodies: Anti-CA and anti-IN for HIV-1 proteins, anti-HSP90 for cytoplasm, anti-calnexin for membranes, anti-HDAC2 for nucleus and anti-histone 3 for chromatin. (B, C) Panels show representative immunoblots probed for HIV-1 CA and IN and the appropriate fractionation marker: (B) Cytoplasm fraction with HSP90 as a loading control, (C) membrane fraction with calnexin as a loading control. “-”indicates uninfected cells. (A, D, E, F, G) Bar charts show the densitometry analysis of the immunoblots plotted as the ratio of CA or IN proteins to the loading control. (A) whole cell lysates, (D) cytoplasm, (E) membranes, (F) nucleus and (G) chromatin fractions. Bar charts show mean ± SEM of at least two independent repeats. The key for bar chart colour coding is shown at the top of figure. (TIF) [file ppat.1009484.s004.tif]

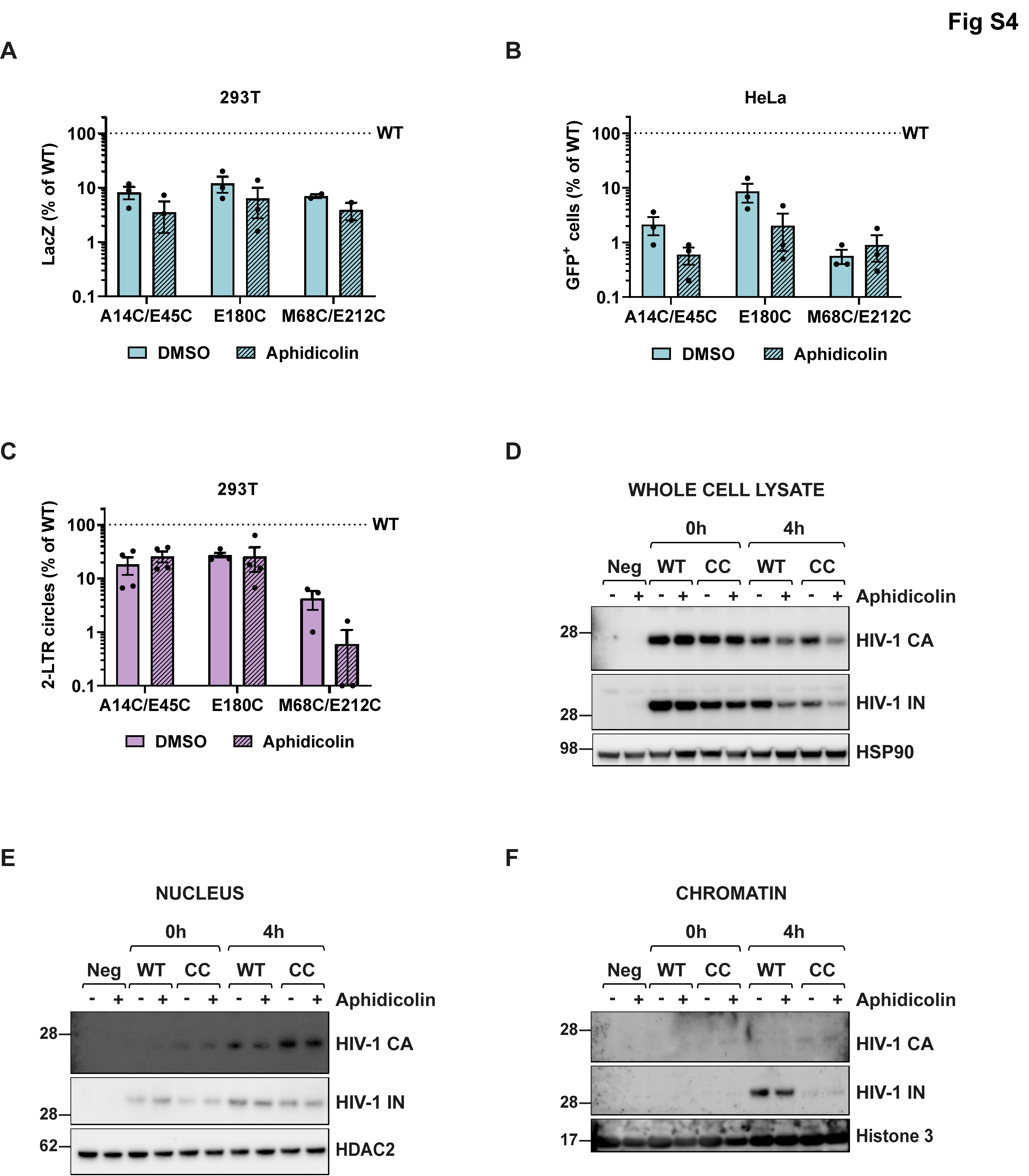

Supplement: S4 Fig — 293T or HeLa cells were treated with either DMSO or aphidicolin at 2μg/ml for 24h, in order to arrest the cells in the G1/S boundary, prior to synchronous infection with WT or mutant VLP. Aphidicolin was maintained in the culture media throughout infection. (A) Treated 293T cells were infected with equal RT units of LacZ-reporter WT or mutant VLP. Cells were lysed at 36hpi and LacZ activity was measured using a chemilumescent assay. The data is shown as % of LacZ activity relative to WT VLP. (B) Treated HeLa cells were infected with equal RT units of GFP-reporter WT or mutant VLP. The percentage of GFP+ cells was measured by flow cytometry at 36hpi and plotted relative to WT VLP. (C) 293T cells were infected with equal RT units of LacZ-reporter WT or mutant VLP. At 24hpi, cells were harvested for DNA extraction and 2-LTR circles were measured by qPCR. All data are plotted relative to WT infections (shown as a dashed line at 100%). Points indicate individual biological repeats and lines show the mean ± SEM. (D-F) Treated HeLa cells were synchronously infected with equal RT units of WT or A14C/E45C mutant (CC) VLP. At 0 (harvested after spinoculation) and 4hpi, cells were harvested in parallel to be processed as a whole cell lysate (WCL) or to undergo subcellular fractionation as in Fig 6. Protein levels were quantified by BCA assay, proportional amounts of the fractions related to the WCL were loaded on SDS-PAGE gels and analysed by immunoblotting for HIV-1 CA or IN and a fractionation marker. Panels show representative immunoblots from two independent experiments of (D) WCL with HSP90 as a loading control, (E) Nuclear fraction with HDAC2 as a loading control, (F) Chromatin fraction with histone3 as a loading control. The first lanes on each blot are uninfected cells (Neg). +/- indicates whether the cells were treated with aphidicolin or DMSO respectively prior to infection. (TIF) [file ppat.1009484.s005.tif]

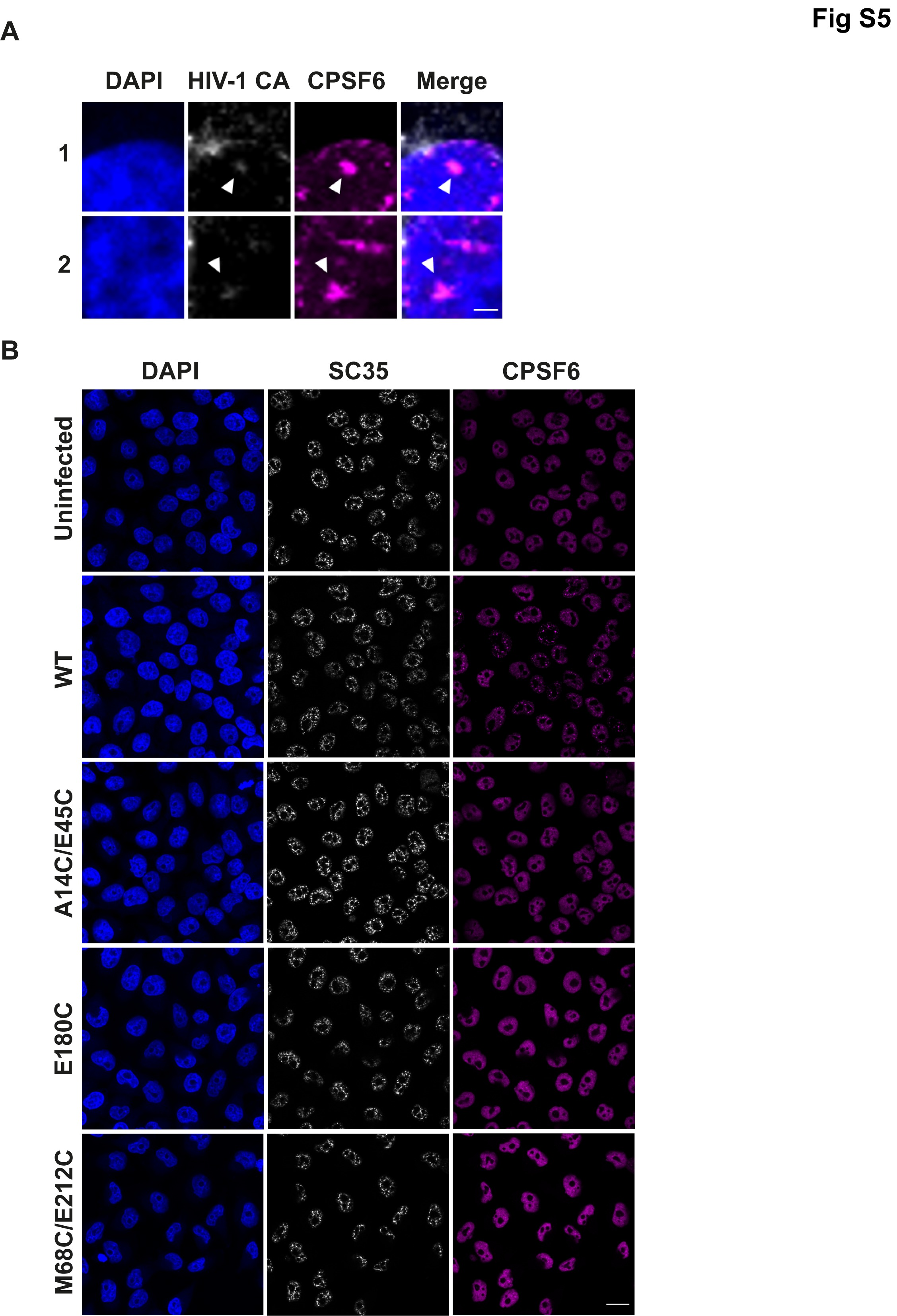

Supplement: S5 Fig — HeLa cells were synchronously infected with equal RT units of WT, A14C/E45C, E180C or M68C/E212C VLP and fixed at 16hpi. Cells were incubated with primary antibodies against HIV-1 CA and CPSF6 (A) or CPSF6 and SC35 (B) followed by specific secondary antibodies conjugated to Alexa Fluor fluorophores. (A) shows zoomed-in images of regions labelled 1 and 2 in Fig 8B. White arrows point to HIV-1 CA and CPSF6 co-localisation. The scale bar is 2 μm. (B) shows representative images of CPSF6 and SC35 staining at a lower magnification (63X) than in Fig 8A. The scale bar is 20 μm. (TIF) [file ppat.1009484.s006.tif]
